# Crystal structures of three copper(II)–2,2′-bi­pyridine (bpy) compounds, [Cu(bpy)_2_(H_2_O)][SiF_6_]·4H_2_O, [Cu(bpy)_2_(TaF_6_)_2_] and [Cu(bpy)_3_][TaF_6_]_2_ and a related coordination polymer, [Cu(bpy)(H_2_O)_2_SnF_6_]_*n*_

**DOI:** 10.1107/S2056989021000633

**Published:** 2021-01-26

**Authors:** Matthew L. Nisbet, Emily Hiralal, Kenneth R. Poeppelmeier

**Affiliations:** a 2145 Sheridan Road, Evanston, IL 60208, USA

**Keywords:** crystal structure, hydro­thermal synthesis, racemic compounds, copper, main group, d^0^ early transition metal

## Abstract

We report the hydro­thermal syntheses and crystal structures of aqua­bis­(2,2′-bi­pyridine-κ^2^ *N*,*N*′)copper(II) hexa­fluorido­silicate tetra­hydrate, [Cu(bpy)_2_(H_2_O)][SiF_6_]·4H_2_O (bpy is 2,2′-bi­pyridine, C_10_H_8_N_2_), (I), bis­(2,2′-bi­pyridine-3κ^2^ *N*,*N*′)-di-μ-fluorido-1:3κ^2^ *F*:*F*;2:3κ^2^ *F*:*F*-deca­fluorido-1κ^5^ *F*,2κ^5^ *F*-ditantalum(V)copper(II), [Cu(bpy)_2_(TaF_6_)_2_], (II), tris­(2,2′-bi­pyridine-κ^2^ *N*,*N*′)copper(II) bis[hexa­fluorido­tantalate(V)], [Cu(bpy)_3_][TaF_6_]_2_, (III), and *catena*-poly[[di­aqua­(2,2′-bi­pyridine-κ^2^ *N*,*N*′)copper(II)]-μ-fluorido-tetra­fluorido­tin-μ-fluorido], [Cu(bpy)(H_2_O)_2_SnF_6_]_*n*_, (IV). Compounds (I), (II) and (III) contain locally chiral copper coordination complexes with *C*_2_, *D*_2_, and *D*_3_ symmetry, respectively. The extended structures of (I) and (IV) are consolidated by O—H⋯F and O—H⋯O hydrogen bonds. The structure of (III) was found to be a merohedral (racemic) twin.

## Abstract 2

The crystal structures of three copper(II)-bi­pyridine–*M*F_6_ (*M* = Si, Ta, Sn) compounds and a related coordination polymer are reported.

## Chemical context   

Copper(II) complexes of 2,2′-bi­pyridine (bpy) adopt a wide range of coordination geometries, including square pyramidal, trigonal bipyramidal and octa­hedral, depending on experimental conditions such as the ligand-to-metal ratio and pH (Garribba *et al.*, 2000 ▸). Previous studies have shown that racemic combinations of chiral [Cu(bpy)_2_(H_2_O)]^2+^ can crystallize in polar structures in the presence of early transition metal fluorides *M*F_6_^2–^, (*M* = Ti, Zr, Hf) (Gautier *et al.*, 2012 ▸; Nisbet *et al.*, 2020 ▸).

Here, we investigate the influence of the anion on the speciation of the copper(II) complex and the arrangement of the ions in the crystal structure in a series of compounds based on copper(II)–2,2′-bi­pyridine cations and SiF_6_^2–^, SnF_6_^2–^ and TaF_6_^−^ anions. Among these hydro­thermally prepared structures we observe three distinct locally chiral copper-bi­pyridine complexes: *C*_2_-symmetric cations in [Cu(bpy)_2_(H_2_O)][SiF_6_]·4H_2_O, (I), *D*_2_-symmetric Cu(bpy)_2_(TaF_6_)_2_ mol­ecules, (II) and *D*_3_-symmetric cations in [Cu(bpy)_3_][TaF_6_]_2_, (III). We also report the structure of a coordination polymer based on Cu(bpy)(H_2_O)_2_^2+^ cations and SnF_6_^2–^ anions, (IV), that forms under similar conditions.

## Structural commentary   

**Figure 1:**
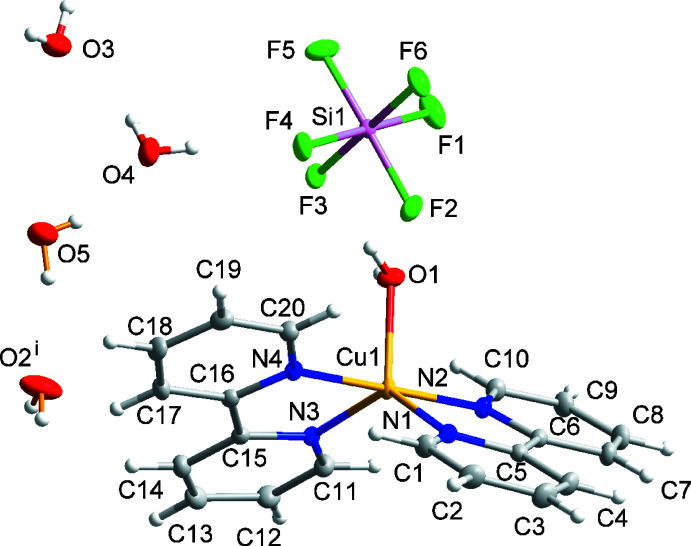
The mol­ecular structure of (I). Ellipsoids of non-H atoms are drawn at 50% probability. H atoms are drawn with an atomic radius of 0.135 Å. [Symmetry code: (i) 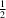 + *x*, 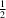 − *y*, 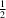 + *z*.]

Compound (I) has the formula [Cu(bpy)_2_(H_2_O)][SiF_6_]·4H_2_O and crystallizes in space group *C*2*/c*. The structure features isolated *C*_2_-symmetric Δ- and Λ-[Cu(bpy)_2_(H_2_O)]^2+^ cations and octa­hedral SiF_6_^2–^ anions (Fig. 1 ▸). The five-coordinate Cu^2+^ ion has a slightly distorted trigonal–bipyramidal coordination environment (τ = 0.77), as described by the parameter τ = (β − α)/60, where β and α are the two largest angles of the complex (τ = 1 corresponds to an ideal trigonal bipyramid and τ = 0 corresponds to an ideal square pyramid) (Melnic *et al.*, 2014 ▸). The average Cu—N bond length and the Cu—OH_2_ bond distance in (I) are in agreement with the reported distances in other known [Cu(bpy)_2_(H_2_O)]^2+^ complexes (Gautier *et al.*, 2012 ▸; Nisbet *et al.*, 2020 ▸; Shi *et al.*, 2010 ▸; Yu *et al.*, 2007 ▸). 
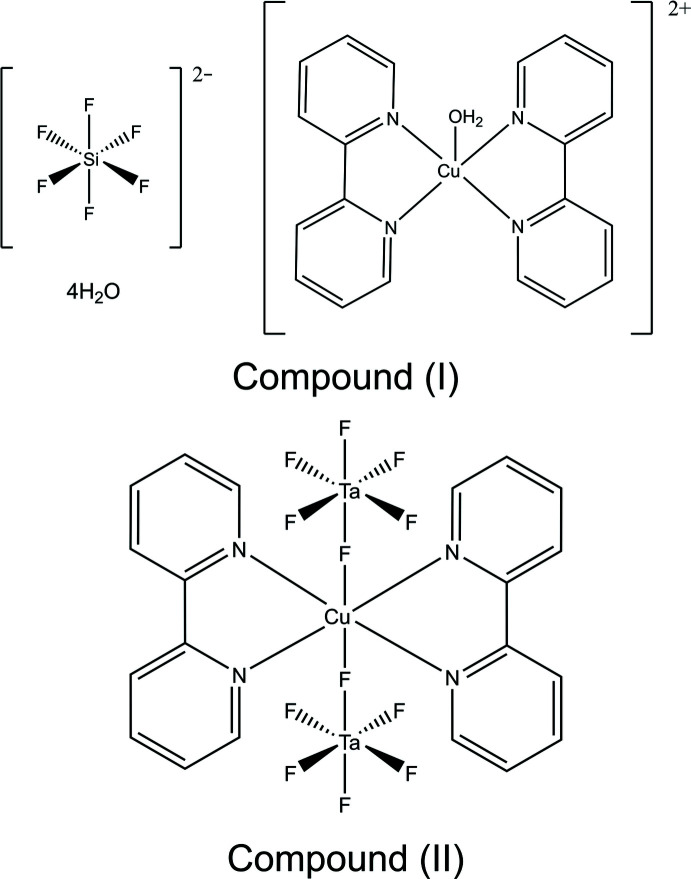
 
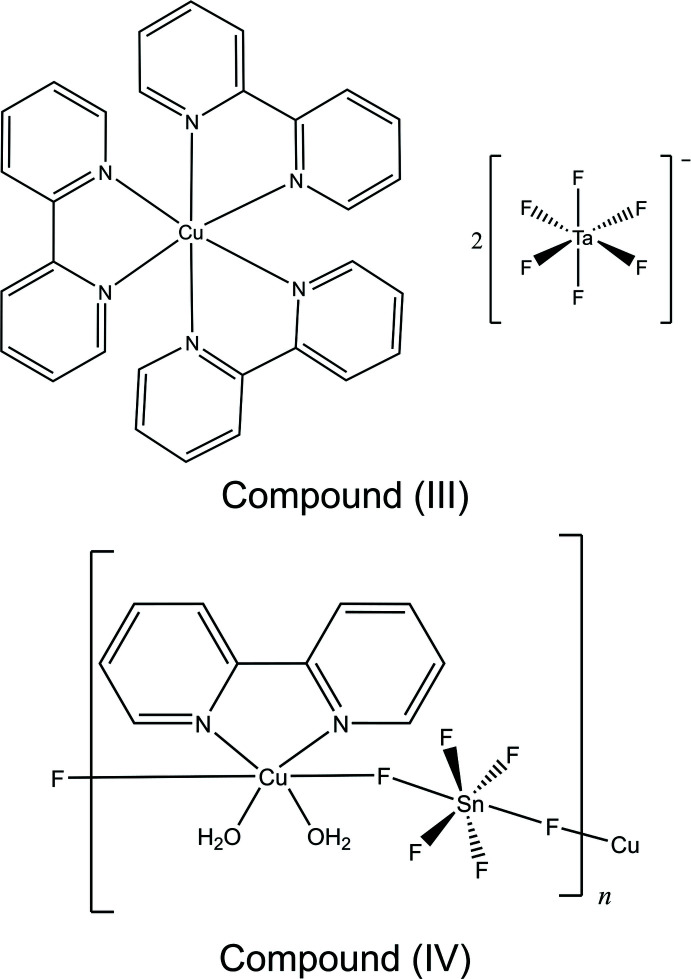


**Figure 2:**
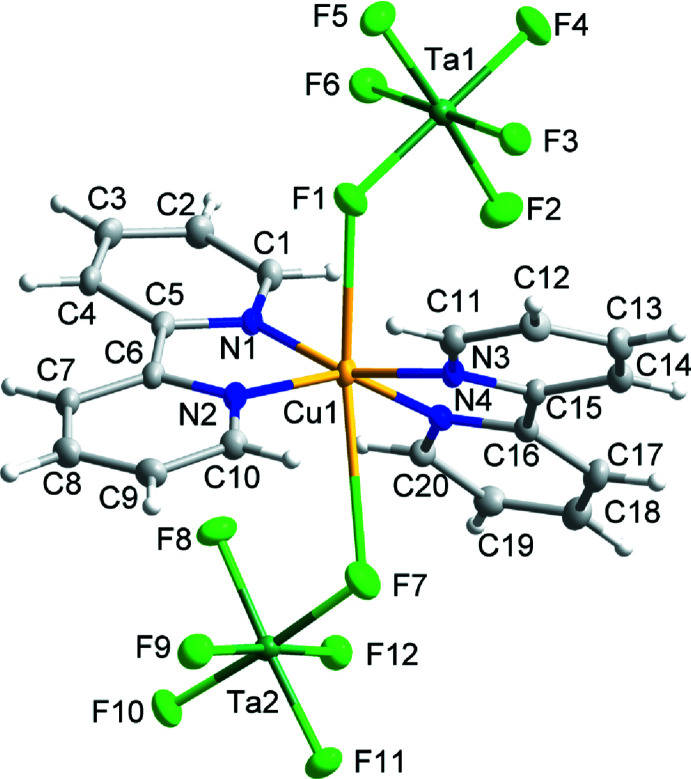
The mol­ecular structure of one of the two independent molecules in (II). Ellipsoids of non-H atoms are drawn at 50% probability. H atoms are drawn with an atomic radius of 0.135 Å.

Compound (II) has the formula Cu(bpy)_2_(TaF_6_)_2_ and crystallizes in space group *P* 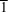. The structure is comprised of mol­ecular Δ- and Λ- Cu(bpy)_2_(TaF_6_)_2_ complexes with local *D*_2_ symmetry. Each Cu^II^ center is equatorially coordinated by two bpy ligands and axially coordinated by two TaF_6_^−^ groups. Two independent Cu(bpy)_2_(TaF_6_)_2_ units with the same handedness are present within the arbitrarily chosen asymmetric unit (Fig. 2 ▸). These complexes differ in their Cu—F bond lengths and F—Cu—F angles: Cu1—F1 = 2.537 (3), Cu1—F7 = 2.987 (3) Å, F1—Cu1—F7 = 161.46 (9)°; Cu2—F13 = 2.706 (3), Cu2—F19 = 2.775 (3) Å, F13—Cu2—F19 = 168.21 (10)°. The observed Cu—F distances fall above the upper quartile of the distribution of known Cu—F bond distances among structures in the Cambridge Structural Database (mean = 2.240 Å, standard deviation = 0.270 Å). The Cu—N and Cu—F distances in (II) are in reasonable agreement with the bond distances reported in the complex (6,6′′′-dimethyl-2,2′:6′,2′′:6′′,2′′′-quaterpyridine)bis­(tetra­fluo­r­o­borate)copper(II) (CSD refcode: UZELOC; Adamski *et al.*, 2017 ▸).

**Figure 3:**
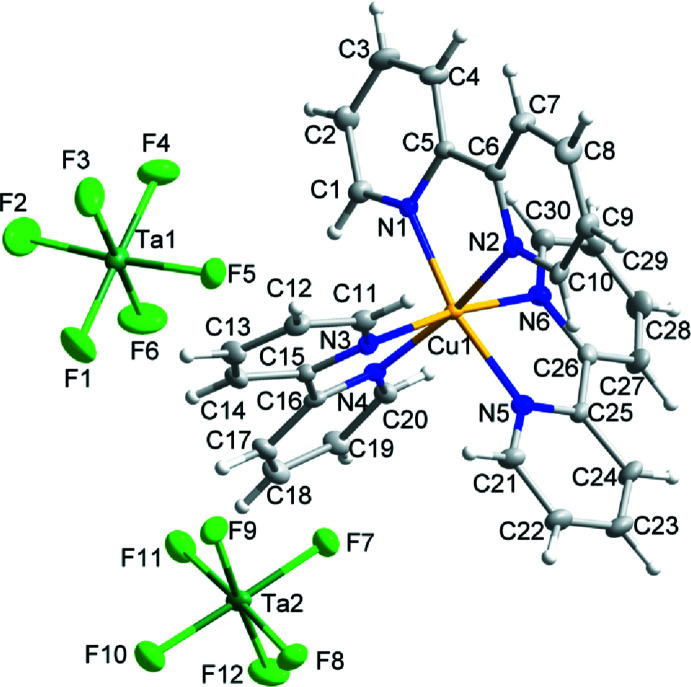
The mol­ecular structure of (III). Ellipsoids of non-H atoms are drawn at 50% probability. H atoms are drawn with an atomic radius of 0.135 Å.

Compound (III) has the formula [Cu(bpy)_3_][TaF_6_]_2_ and crystallizes in the enanti­omorphous space group *P*3_2_. The structure of (III) contains *D*_3_-symmetric Λ-Cu(bpy)_3_^2+^ cations with Cu^II^ in an octa­hedral CuN_6_ coordination environment. The Cu—N distances are in agreement with those of the Cu(bpy)_3_^2+^ cations in [Cu(bpy)_3_][PF_6_]_2_ (CSD refcode: REZJAI; Wang *et al.*, 2007 ▸) and [Cu(bpy)_3_][BF_4_]_2_ (CSD refcode: RIGTEH; Chamayou *et al.*, 2007 ▸). Two distinct octa­hedral TaF_6_^−^ anions are present in the asymmetric unit (Fig. 3 ▸).

**Figure 4:**
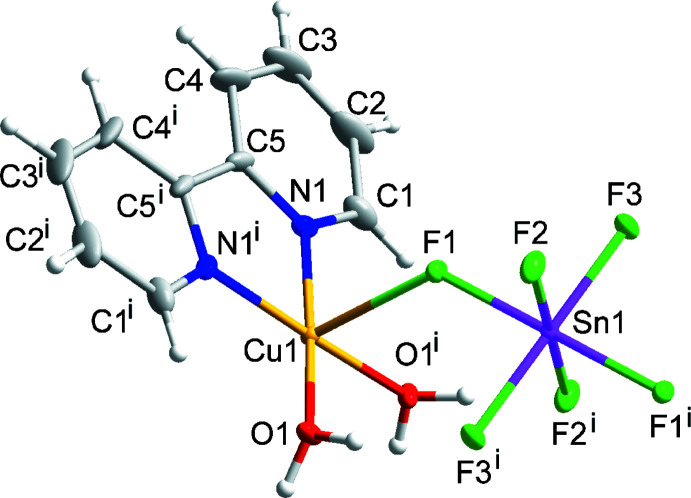
The mol­ecular structure of (IV). Ellipsoids of non-H atoms are drawn at 50% probability. H atoms are drawn with an atomic radius of 0.135 Å. [Symmetry code: (i) 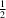 − *x*, *y*, 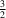 − *z*.]

Compound (IV) has the formula Cu(bpy)(H_2_O)_2_SnF_6_ and crystallizes in space group *P*2*/n*. The structure is composed of one-dimensional coordination chains propagating in the [101] direction that can be described as alternating Cu(bpy)(H_2_O)_2_^2+^ cations (Cu site symmetry 2) and SnF_6_^2−^ anions catenated through bridging Cu—F—Sn linkages. The Sn^4+^ ion occupies a crystallographic inversion center. Intra­molecular hydrogen bonding is present along the chains *via* O1—H1*A*⋯F2 and O1—H1*B*⋯F3 contacts (Fig. 4 ▸; Table 2 ▸). The Cu—F bond distance of 2.3830 (10) Å is in agreement with those found in the reported compound Cu(4,4′-bi­pyridine)_2_SiF_6_ (CSD refcode: PETWES; Nugent *et al.*, 2013 ▸).

## Supra­molecular features   

**Figure 5:**
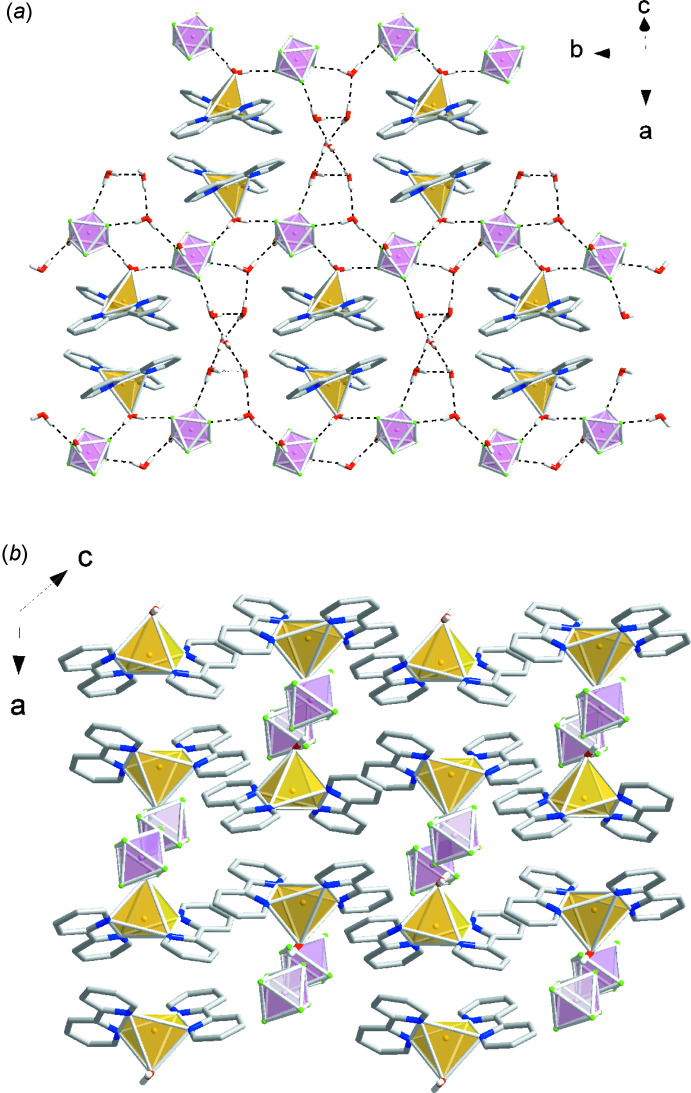
Packing diagram for (I): yellow polyhedra represent Cu(bpy)_2_(H_2_O)^2+^ cations and pink polyhedra represent SiF_6_^2−^ anions.

In the extended structure of (I), the Cu(bpy)_2_(H_2_O)^2+^ and SiF_6_^2−^ groups are linked *via* O—H⋯F hydrogen bonding between the apical water mol­ecule and two SiF_6_^2−^ ions (Table 1 ▸). The Δ/Λ-Cu(bpy)_2_(H_2_O)^2+^ units participate in displaced heterochiral π–π stacking inter­actions between the N1/C1–C5 and N2/C6–C10 rings with an inter­planar angle of 1.11 (11)°, centroid–centroid distance of 3.8774 (12) Å, and a slippage distance of 1.490 Å to form Δup–Λdown–Δup–Λdown and Δdown–Λup–Δdown–Λup chains (up/down refers to the orientation of the Cu—O bond vector in the +*a* or –*a* direction). The water mol­ecules of hydration are involved in O—H⋯F hydrogen bonding inter­actions with the SiF_6_^2−^ anion as well as O—H⋯O bonds with other water mol­ecules (Fig. 5 ▸).

**Figure 6:**
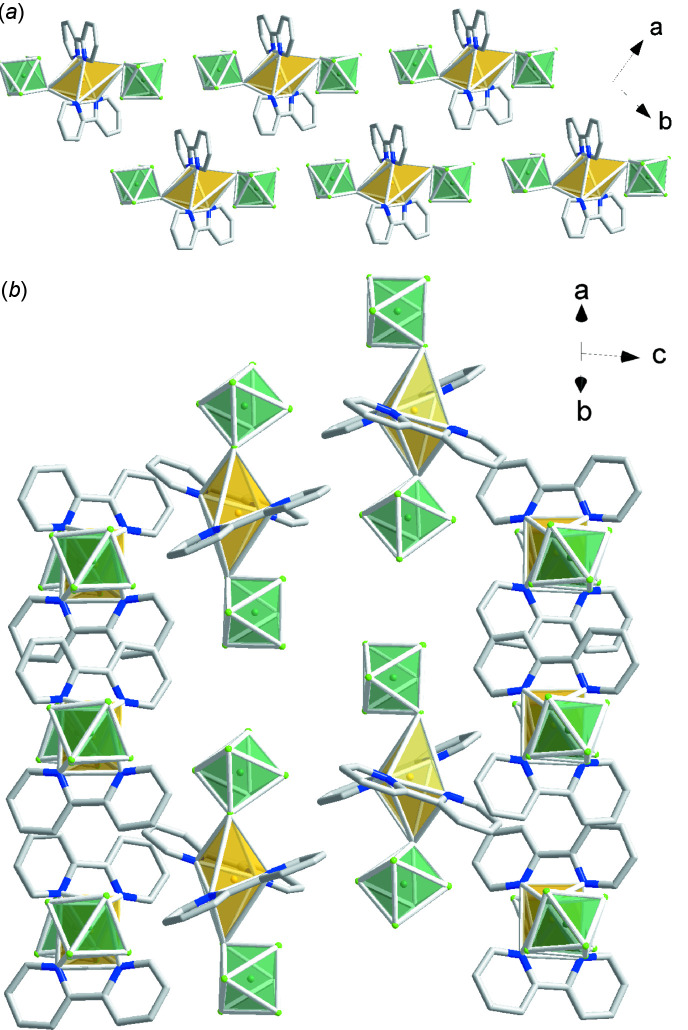
Packing diagram for (II): yellow polyhedra represent Cu(bpy)_2_^2+^ cations and green polyhedra represent TaF_6_^−^ anions.

The neutral Cu(bpy)_2_(TaF_6_)_2_ complexes in (II) form homochiral chains in which the F—Cu—F bond axes of adjacent complexes are aligned along the *a* + *b* or *b – a* directions, as shown in Fig. 6 ▸. Along the *c*-axis direction, each chain is neighbored by a chain with the opposite chirality and same orientation on one side and a chain with the same chirality and opposite orientation on the other.

**Figure 7:**
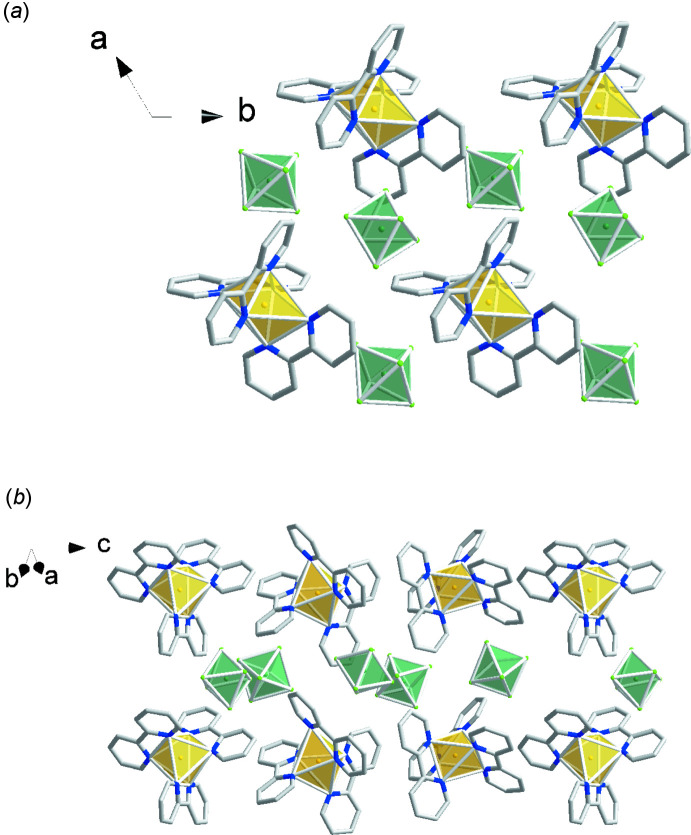
Packing diagram for (III): yellow polyhedra represent Cu(bpy)_3_^2+^ cations and green polyhedra represent TaF_6_^−^ anions.

In (III), the Λ-Cu(bpy)_3_^2+^ complexes participate in displaced π–π stacking inter­actions propagating along the 3_2_ screw axes with an inter­planar angle of 13.9 (2)°, centroid–centroid distance of 3.933 (2) Å between adjacent N1/C1–C5 and N5/C21–C25 pyridine rings, and a horizontal shift distance of 1.970 Å. Each Λ-Cu(bpy)_3_^2+^ cation is surrounded by six TaF_6_^−^ anions (Fig. 7 ▸).

**Figure 8:**
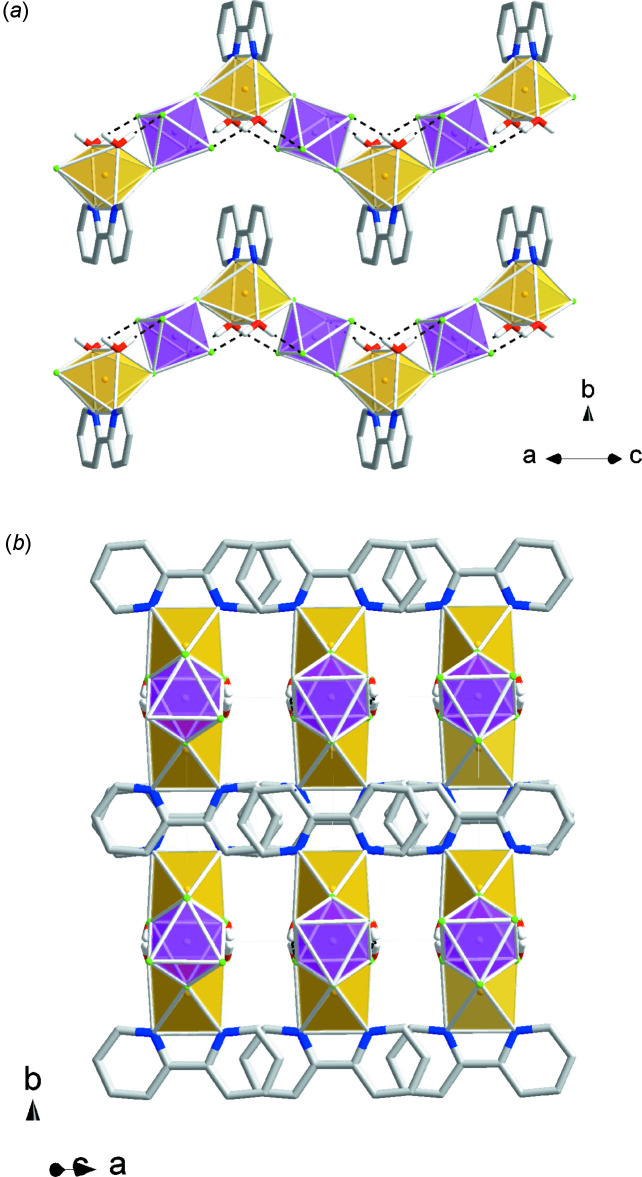
Packing diagram for (IV): Yellow polyhedra represent Cu(bpy)(H_2_O)_2_^2+^ cations and magenta polyhedra represent SnF_6_^2−^ anions.

The one-dimensional coordination chains in (IV) pack in a brickwork arrangement *via* parallel displaced π–π stacking inter­actions (Fig. 8 ▸). One of the stacking inter­actions involves parallel N1/C1–C5 pyridine rings at a centroid–centroid distance of 3.8133 (12) Å and a shift distance 1.676 Å, while the other stacking inter­action involves nonparallel N1/C1–C5 pyridine rings with an inter­planar angle of 3.54 (11)°, centroid–centroid distance of 3.5830 (14) Å and a shift distance of 1.072 Å.

## Database survey   

A survey of structures related to (I) reported in the Cambridge Structural Database (CSD, version 2020.2.0 from September 2020; Groom *et al.*, 2016 ▸) produced five other compounds based on [Cu(bpy)_2_(H_2_O)]^2+^ complexes and fluorinated inorganic anions: [Cu(bpy)_2_(H_2_O)][BF_4_]_2_ (CSD refcode: VIKDOJ; Yu *et al.*, 2007 ▸), [Cu(bpy)_2_(H_2_O)][PF_6_]_2_ (CSD refcode: EQIQOL; Shi *et al.*, 2010 ▸), and [Cu(bpy)_2_(H_2_O)][*M*F_6_] (*M* = Ti, Zr, Hf; CSD refcodes: GESHOD, YUGYEH, YUGYIL, YUGYOR; Gautier *et al.*, 2012 ▸; Nisbet *et al.*, 2020 ▸). These compounds display a variety of packing architectures, with compounds based on singly charged PF_6_^−^ and BF_4_^−^ anions displaying hydrogen-bonded clusters composed of two anions and one cation while compounds based on doubly charged *M*F_6_^2−^ anions form extended hydrogen-bonded networks. The hydrogen-bonding inter­actions in (I) differ from the analogous compounds based on early transition-metal fluorides in that the *M*F_6_^2−^ anions hydrogen bonded to the [Cu(bpy)_2_(H_2_O)]^2+^ complex are both hydrogen bonded to the same pair of [Cu(bpy)_2_)(H_2_O)]^2+^ complexes in the ETM case, whereas they are bound to two different complexes in the SiF_6_^2−^ case. Further, while the [Cu(bpy)_2_(H_2_O)][*M*F_6_] (*M* = Ti, Zr, Hf) compounds display both face-to-face and displaced π–π stacking inter­actions, (I) has only displaced stacking inter­actions.

A search of the CSD for structures related to (II) revealed no other known octa­hedral bis­(2,2′-bi­pyridine)­copper(II) complexes with two fluorinated anions coordinated in the apical positions. The most similar example known to the authors is (6,6′′′-dimethyl-2,2′:6′,2′′:6′′,2′′′-quaterpyridine)­bis­(tetra­fluoro­borate)copper(II) (CSD refcode: UZELOC; Adamski *et al.*, 2017 ▸). This structure features copper(II) complexes arranged such that the F—Cu—F axis of each complex is oriented along the *a*-axis direction. Additionally, these complexes participate in heterochiral π–π stacking inter­actions.

Compound (III) is a new member of the family of compounds that includes [*A*(bpy)_3_][PF_6_] (*A* = Mn, Co, Ni, Cu, Zn, Ru, and Cd; CSD refcodes: YEGLUR, VUMTEE, WOTSAZ01, REZJAI, WOTSON, BPYRUG, XEFNOM, respectively; (Deisenroth *et al.*, 2001 ▸); Breu *et al.*, 2000 ▸; Björemark *et al.*, 2015 ▸; Wang *et al.*, 2007 ▸; Kundu *et al.*, 2005 ▸), Zn(bpy)_3_][TaF_6_]_2_ (CSD refcode: HAHFII; Gautier & Poeppelmeier, 2016 ▸), and [Zn(bpy)_3_][NbF_6_]_2_ (CSD refcode: HAHFUU; Gautier & Poeppelmeier, 2016 ▸). These compounds include either Δ- or Λ-Cu(bpy)_3_^2+^ cations arranged along 3_1_ or 3_2_ screw axes depending on the handedness of the Cu(bpy)_3_^2+^ complexes.

Compound (IV) is isostructural to the coordination polymer Cu(bpy)(H_2_O)HfF_6_ (CSD refcode: YUGXOQ; Nisbet *et al.*, 2020 ▸). These compounds share identical connectivity with a series of coordination polymers with the formula *M*′(bpy)(H_2_O)_2_ *M*O_*x*_F_6–*x*_ compounds (*M*′/*M* = Cu/Ti, Cu/V, Cu/Nb, Cu/Mo, Zn/Mo, and Zn/W), which display polar zigzag chains (Gautier & Poeppelmeier, 2013 ▸).

## Synthesis and crystallization   

The compounds reported here were synthesized by the hydro­thermal pouch method (Harrison *et al.*, 1993 ▸). In each reaction, reagents were heat sealed in Teflon pouches. Groups of six pouches were then placed into a 125 ml Parr autoclave with 40 ml distilled water as backfill. The autoclave was heated at a rate of 5°C min^−1^ to 150°C and held at 150°C for 24 h. The autoclaves were allowed to cool to room temperature at a rate of 6°C h^−1^ and the solid products were recovered by vacuum filtration. Compound (I) was synthesized in a pouch containing 1.9 mmol of Cu(NO_3_)_2_·H_2_O, 5 mmol of 2,2′-bi­pyridine, 1.5 mmol of (NH_4_)_2_SiF_6_ and 1ml of deionized H_2_O. Compound (II) was synthesized in a pouch containing 1.7 mmol of CuO, 2.5 mmol of 2,2′-bi­pyridine, 0.85 mmol of Ta_2_O_5_, 0.8 ml HF(aq), and 0.3 ml of deionized H_2_O. Compound (III) was synthesized in a pouch containing 1.7 mmol of CuO, 5.1 mmol of 2,2′-bi­pyridine, 0.85 mmol Ta_2_O_5_, 1 ml of HF(aq) and 0.1 ml of deionized H_2_O. Compound (IV) was synthesized in a pouch containing 1.9 mmol of Cu(NO_3_)_2_·H_2_O, 1.3 mmol of 2,2′-bi­pyridine, 1.7 mmol of (NH_4_)_2_SnF_6_ and 1 ml of deionized H_2_O.

## Refinement   

Crystal data, data collection and structure refinement details are summarized in Table 3 ▸. Hydrogen atom positions were assigned from difference map peaks and their positions freely refined with the exception of C—H hydrogen atoms of 2,2′-bi­pyridine, which were constrained to ride at distances of 0.95 Å from the associated C atoms with *U*_iso_(H) = 1.2*U*_eq_(C).

The measured crystal of (III) is a class II twin by merohedry about a twofold axis along the [110] direction to give apparent Laue symmetry of 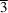 *m*1. The twinning occurs with a BASF of 0.5, suggesting that both the *P*3_1_ and *P*3_2_ configurations are present in equal proportions within the sample.

## Supplementary Material

Crystal structure: contains datablock(s) global, II, III, IV, I. DOI: 10.1107/S2056989021000633/hb7957sup1.cif

CCDC references: 2048914, 2048918, 2048917, 2048915

Additional supporting information:  crystallographic information; 3D view; checkCIF report

## Figures and Tables

**Table 1:** Hydrogen-bond geometry (Å, °) for (I)

*D*—H⋯*A*	*D*—H	H⋯*A*	*D*⋯*A*	*D*—H⋯*A*
O1—H1*A*⋯F3	0.76 (3)	1.91 (3)	2.6616 (14)	177 (3)
O1—H1*B*⋯F6^i^	0.78 (2)	1.93 (2)	2.7053 (14)	170 (2)
O2—H2*A*⋯F1	0.75 (2)	1.94 (2)	2.6677 (17)	164 (2)
O2—H2*B*⋯F4^i^	0.79 (3)	2.00 (3)	2.7807 (17)	167 (2)
O3—H3*A*⋯F5^ii^	0.77 (3)	1.99 (3)	2.7607 (18)	177 (3)
O3—H3*B*⋯O5^iii^	0.73 (3)	2.06 (3)	2.779 (2)	171 (3)
O4—H4*A*⋯O3	0.72 (2)	2.05 (2)	2.749 (2)	162 (2)
O4—H4*B*⋯F4	0.77 (3)	1.98 (3)	2.7462 (16)	170 (2)
O5—H5*A*⋯O4	0.69 (2)	2.13 (2)	2.779 (2)	158 (2)
O5—H5*B*⋯O2^iv^	0.81 (3)	1.99 (3)	2.786 (2)	169 (2)

Symmetry codes: (i) 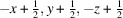; (ii) 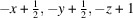; (iii) 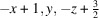; (iv) 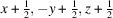.

**Table 2:** Hydrogen-bond geometry (Å, °) for (IV)

*D*—H⋯*A*	*D*—H	H⋯*A*	*D*⋯*A*	*D*—H⋯*A*
O1—H1*A*⋯F2^i^	0.88 (3)	1.79 (3)	2.6444 (17)	165 (3)
O1—H1*B*⋯F3^ii^	0.81 (3)	1.84 (4)	2.6293 (17)	164 (4)

Symmetry codes: (i) 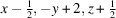; (ii) 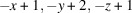.

**Table 3:** Experimental details

	(I)	(II)	(III)	(IV)
Crystal data				
Chemical formula	[Cu(C_10_H_8_N_2_)_2_(H_2_O)][SiF_6_]·4H_2_O	[CuTa_2_F_12_(C_10_H_8_N_2_)_2_]	[Cu(C_10_H_8_N_2_)_3_][TaF_6_]_2_	[CuSnF_6_(C_10_H_8_N_2_)(H_2_O)_2_]
*M*_r_	608.08	965.81	1121.99	488.45
Crystal system, space group	Monoclinic, *C*2/*c*	Triclinic, *P* 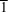	Trigonal, *P*3_2_	Monoclinic, *P*2/*n*
Temperature (K)	100	100	100	100
*a*, *b*, *c* (Å)	25.4971 (16), 13.3573 (9), 18.944 (2)	9.5465 (1), 10.5102 (1), 25.9853 (4)	10.5172 (10), 10.5172 (10), 26.288 (2)	6.2590 (2), 9.2167 (3), 12.1648 (3)
α, β, γ (°)	90, 131.949 (1), 90	96.723 (1), 100.256 (1), 96.672 (1)	90, 90, 120	90, 90.734 (2), 90
*V* (Å^3^)	4798.5 (7)	2522.78 (5)	2518.2 (5)	701.70 (4)
*Z*	8	4	3	2
Radiation type	Mo *K*α	Mo *K*α	Mo *K*α	Mo *K*α
μ (mm^−1^)	1.05	9.60	7.23	3.37
Crystal size (mm)	0.30 × 0.26 × 0.15	0.52 × 0.32 × 0.22	0.22 × 0.16 × 0.12	0.20 × 0.13 × 0.12

Data collection				
Diffractometer	Bruker APEXII CCD	Rigaku Oxford Diffraction XtaLAB Synergy, Single source at offset/far, HyPix	Bruker Kappa APEX CCD area detector	Rigaku Oxford Diffraction XtaLAB Synergy, Single source at offset/far, HyPix
Absorption correction	Multi-scan (*SADABS*; Bruker, 2016 ▸)	Gaussian *CrysAlis PRO* (Rigaku OD, 2020 ▸)	Multi-scan (*SADABS*; Bruker, 2016 ▸)	Gaussian *CrysAlis PRO* (Rigaku OD, 2020 ▸)
*T*_min_, *T*_max_	0.694, 0.746	0.035, 0.414	0.559, 0.746	0.732, 1.000
No. of measured, independent and observed [*I* > 2σ(*I*)] reflections	57770, 6660, 5863	93570, 18263, 15170	148130, 12260, 12121	22131, 3686, 3251
*R*_int_	0.050	0.060	0.050	0.055
(sin θ/λ)_max_ (Å^−1^)	0.693	0.785	0.761	0.870

Refinement				
*R*[*F* ^2^ > 2σ(*F* ^2^)], *wR*(*F* ^2^), *S*	0.027, 0.070, 1.04	0.037, 0.088, 1.05	0.016, 0.031, 1.03	0.029, 0.067, 1.07
No. of reflections	6660	18263	12260	3686
No. of parameters	374	704	462	110
No. of restraints	0	0	1	0
H-atom treatment	H atoms treated by a mixture of independent and constrained refinement	H-atom parameters constrained	H-atom parameters constrained	H atoms treated by a mixture of independent and constrained refinement
Δρ_max_, Δρ_min_ (e Å^−3^)	0.79, −0.25	1.67, −3.53	0.96, −0.85	2.05, −0.85
Absolute structure	–	–	Flack *x* determined using 5861 quotients [(*I* ^+^)−(*I* ^−^)]/[(*I* ^+^)+(*I* ^−^)] (Parsons *et al.*, 2013 ▸)	–
Absolute structure parameter	–	–	0.5036 (7)	–

Computer programs: *APEX2* (Bruker, 2017 ▸), *SAINT* (Bruker, 2016 ▸), *CrysAlis PRO* (Rigaku OD, 2020 ▸), *SHELXT* (Sheldrick, 2015*a* ▸), *SHELXL* (Sheldrick, 2015*b* ▸), and *OLEX2* (Dolomanov *et al.*, 2009 ▸).

## supplementary crystallographic information

### Aquabis(2,2'-bipyridine-κ2N,N')copper(II) hexafluoridosilicate tetrahydrate (I) Crystal data

**Table:**

[Cu(C_10_H_8_N_2_)_2_(H_2_O)][SiF_6_]·4H_2_O	*F*(000) = 2488
*M**_r_* = 608.08	*D*_x_ = 1.683 Mg m^−^^3^
Monoclinic, *C*2/*c*	Mo *K*α radiation, λ = 0.71073 Å
*a* = 25.4971 (16) Å	Cell parameters from 9731 reflections
*b* = 13.3573 (9) Å	θ = 2.8–29.4°
*c* = 18.944 (2) Å	µ = 1.05 mm^−^^1^
β = 131.949 (1)°	*T* = 100 K
*V* = 4798.5 (7) Å^3^	Block, blue
*Z* = 8	0.30 × 0.26 × 0.15 mm

### Aquabis(2,2'-bipyridine-κ2N,N')copper(II) hexafluoridosilicate tetrahydrate (I) Data collection

**Table:**

Bruker APEXII CCD diffractometer	6660 independent reflections
Radiation source: sealed tube	5863 reflections with *I* > 2σ(*I*)
Triumph monochromator	*R*_int_ = 0.050
Detector resolution: 8 pixels mm^-1^	θ_max_ = 29.5°, θ_min_ = 1.9°
φ and ω scans	*h* = −35→34
Absorption correction: multi-scan (SADABS; Bruker, 2016)	*k* = −18→18
*T*_min_ = 0.694, *T*_max_ = 0.746	*l* = −24→26
57770 measured reflections	

### Aquabis(2,2'-bipyridine-κ2N,N')copper(II) hexafluoridosilicate tetrahydrate (I) Refinement

**Table:**

Refinement on *F*^2^	Primary atom site location: dual
Least-squares matrix: full	Hydrogen site location: mixed
*R*[*F*^2^ > 2σ(*F*^2^)] = 0.027	H atoms treated by a mixture of independent and constrained refinement
*wR*(*F*^2^) = 0.070	*w* = 1/[σ^2^(*F*_o_^2^) + (0.0277*P*)^2^ + 6.982*P*] where *P* = (*F*_o_^2^ + 2*F*_c_^2^)/3
*S* = 1.04	(Δ/σ)_max_ = 0.001
6660 reflections	Δρ_max_ = 0.79 e Å^−^^3^
374 parameters	Δρ_min_ = −0.24 e Å^−^^3^
0 restraints	

### Aquabis(2,2'-bipyridine-κ2N,N')copper(II) hexafluoridosilicate tetrahydrate (I) Special details

**Table:**

Geometry. All esds (except the esd in the dihedral angle between two l.s. planes) are estimated using the full covariance matrix. The cell esds are taken into account individually in the estimation of esds in distances, angles and torsion angles; correlations between esds in cell parameters are only used when they are defined by crystal symmetry. An approximate (isotropic) treatment of cell esds is used for estimating esds involving l.s. planes.

### Aquabis(2,2'-bipyridine-κ2N,N')copper(II) hexafluoridosilicate tetrahydrate (I) Fractional atomic coordinates and isotropic or equivalent isotropic displacement parameters (Å^2^)

**Table:**

	*x*	*y*	*z*	*U*_iso_*/*U*_eq_	
Cu1	0.38373 (2)	0.37571 (2)	0.22886 (2)	0.01115 (5)	
O1	0.30718 (6)	0.41614 (9)	0.23571 (8)	0.0177 (2)	
H1A	0.3036 (13)	0.3839 (18)	0.2650 (17)	0.035 (6)*	
H1B	0.3081 (12)	0.4726 (19)	0.2479 (16)	0.033 (6)*	
N1	0.37806 (6)	0.26410 (8)	0.15033 (8)	0.0120 (2)	
N3	0.46003 (6)	0.48439 (9)	0.31770 (8)	0.0122 (2)	
N4	0.44106 (6)	0.30358 (8)	0.35115 (8)	0.0124 (2)	
N2	0.32619 (6)	0.44426 (9)	0.10503 (8)	0.0124 (2)	
C6	0.31103 (7)	0.39110 (10)	0.03265 (9)	0.0117 (2)	
C16	0.49954 (7)	0.35110 (10)	0.42717 (9)	0.0122 (2)	
C15	0.51060 (7)	0.45257 (10)	0.40829 (9)	0.0120 (2)	
C17	0.54345 (8)	0.30770 (11)	0.51640 (10)	0.0164 (3)	
H17	0.584712	0.341450	0.569103	0.020*	
C4	0.33051 (8)	0.22242 (11)	−0.00601 (10)	0.0166 (3)	
H4	0.303744	0.241407	−0.070316	0.020*	
C5	0.34040 (7)	0.28885 (10)	0.05837 (9)	0.0120 (2)	
C11	0.46479 (8)	0.57742 (10)	0.29641 (10)	0.0156 (3)	
H11	0.429070	0.600245	0.233009	0.019*	
C18	0.52625 (8)	0.21425 (11)	0.52759 (10)	0.0181 (3)	
H18	0.555859	0.183054	0.588184	0.022*	
C12	0.51911 (8)	0.64187 (11)	0.36212 (11)	0.0177 (3)	
H12	0.520466	0.707626	0.344271	0.021*	
C20	0.42436 (8)	0.21398 (10)	0.36308 (10)	0.0149 (3)	
H20	0.382534	0.181730	0.309805	0.018*	
C14	0.56701 (8)	0.51293 (11)	0.47781 (10)	0.0168 (3)	
H14	0.602223	0.488785	0.540838	0.020*	
C19	0.46602 (8)	0.16686 (11)	0.45043 (10)	0.0165 (3)	
H19	0.453313	0.103009	0.457052	0.020*	
C10	0.30008 (8)	0.53675 (10)	0.08876 (10)	0.0151 (3)	
H10	0.309292	0.572663	0.139338	0.018*	
C3	0.36042 (9)	0.12784 (11)	0.02521 (11)	0.0197 (3)	
H3	0.353934	0.080777	−0.017661	0.024*	
C2	0.39978 (9)	0.10290 (11)	0.11951 (11)	0.0192 (3)	
H2	0.421146	0.038806	0.142568	0.023*	
C9	0.26007 (8)	0.58174 (11)	0.00054 (10)	0.0165 (3)	
H9	0.242705	0.647922	−0.009131	0.020*	
C13	0.57152 (8)	0.60901 (11)	0.45437 (11)	0.0187 (3)	
H13	0.609914	0.651426	0.500877	0.022*	
C7	0.27095 (8)	0.43169 (11)	−0.05742 (10)	0.0159 (3)	
H7	0.260819	0.393583	−0.107643	0.019*	
C1	0.40748 (8)	0.17297 (10)	0.17963 (10)	0.0158 (3)	
H1	0.434783	0.155761	0.244450	0.019*	
C8	0.24582 (8)	0.52859 (11)	−0.07328 (10)	0.0178 (3)	
H8	0.219083	0.558236	−0.134231	0.021*	
Si1	0.23943 (2)	0.20875 (3)	0.28568 (3)	0.01040 (8)	
F4	0.30094 (5)	0.12511 (7)	0.36761 (6)	0.02014 (19)	
F2	0.26224 (5)	0.18737 (8)	0.22236 (6)	0.0246 (2)	
F6	0.17998 (5)	0.11481 (6)	0.22816 (6)	0.02098 (19)	
F3	0.30005 (5)	0.30181 (7)	0.34414 (6)	0.01928 (18)	
F5	0.21850 (6)	0.23088 (7)	0.35102 (8)	0.0248 (2)	
F1	0.17872 (5)	0.29058 (7)	0.20409 (7)	0.0281 (2)	
O2	0.14362 (8)	0.48395 (10)	0.17503 (12)	0.0332 (3)	
H2A	0.1591 (11)	0.4330 (17)	0.1834 (15)	0.023 (5)*	
H2B	0.1657 (13)	0.5213 (19)	0.1710 (17)	0.036 (6)*	
O3	0.39995 (8)	0.19743 (10)	0.68756 (10)	0.0252 (3)	
H3A	0.3668 (14)	0.2152 (18)	0.6774 (17)	0.037 (7)*	
H3B	0.4124 (14)	0.156 (2)	0.7206 (19)	0.046 (8)*	
O4	0.41008 (7)	0.12569 (10)	0.56108 (9)	0.0246 (3)	
H4A	0.3995 (12)	0.1479 (17)	0.5847 (16)	0.028 (6)*	
H4B	0.3776 (14)	0.1313 (17)	0.5077 (19)	0.036 (7)*	
O5	0.54187 (8)	0.03556 (10)	0.68866 (10)	0.0267 (3)	
H5A	0.5100 (11)	0.0531 (15)	0.6479 (15)	0.013 (5)*	
H5B	0.5669 (13)	0.0274 (18)	0.6773 (17)	0.040 (7)*	

### Aquabis(2,2'-bipyridine-κ2N,N')copper(II) hexafluoridosilicate tetrahydrate (I) Atomic displacement parameters (Å^2^)

**Table:**

	*U*^11^	*U*^22^	*U*^33^	*U*^12^	*U*^13^	*U*^23^
Cu1	0.01351 (9)	0.00970 (8)	0.00754 (8)	0.00123 (6)	0.00592 (7)	0.00124 (5)
O1	0.0241 (6)	0.0120 (5)	0.0260 (6)	0.0011 (4)	0.0205 (5)	0.0028 (4)
N1	0.0122 (6)	0.0115 (5)	0.0113 (5)	0.0010 (4)	0.0075 (5)	0.0002 (4)
N3	0.0126 (6)	0.0127 (5)	0.0104 (5)	−0.0005 (4)	0.0074 (5)	0.0006 (4)
N4	0.0132 (6)	0.0124 (5)	0.0110 (5)	0.0015 (4)	0.0078 (5)	0.0019 (4)
N2	0.0138 (6)	0.0115 (5)	0.0103 (5)	0.0019 (4)	0.0074 (5)	0.0009 (4)
C6	0.0118 (6)	0.0125 (6)	0.0107 (6)	−0.0002 (5)	0.0074 (5)	0.0001 (5)
C16	0.0122 (6)	0.0132 (6)	0.0121 (6)	0.0020 (5)	0.0085 (5)	0.0014 (5)
C15	0.0117 (6)	0.0136 (6)	0.0115 (6)	0.0010 (5)	0.0080 (5)	0.0006 (5)
C17	0.0128 (7)	0.0194 (7)	0.0114 (6)	0.0012 (5)	0.0058 (6)	0.0015 (5)
C4	0.0212 (8)	0.0165 (6)	0.0141 (6)	0.0005 (5)	0.0126 (6)	−0.0010 (5)
C5	0.0116 (6)	0.0122 (6)	0.0118 (6)	0.0000 (5)	0.0078 (5)	0.0004 (5)
C11	0.0179 (7)	0.0141 (6)	0.0144 (6)	−0.0008 (5)	0.0105 (6)	0.0016 (5)
C18	0.0171 (7)	0.0211 (7)	0.0134 (6)	0.0056 (5)	0.0090 (6)	0.0078 (5)
C12	0.0206 (7)	0.0137 (6)	0.0201 (7)	−0.0039 (5)	0.0141 (6)	−0.0014 (5)
C20	0.0151 (7)	0.0151 (6)	0.0142 (6)	0.0003 (5)	0.0097 (6)	0.0015 (5)
C14	0.0145 (7)	0.0189 (7)	0.0130 (6)	−0.0010 (5)	0.0075 (6)	−0.0011 (5)
C19	0.0193 (7)	0.0146 (6)	0.0191 (7)	0.0033 (5)	0.0142 (6)	0.0049 (5)
C10	0.0170 (7)	0.0129 (6)	0.0138 (6)	0.0017 (5)	0.0097 (6)	0.0004 (5)
C3	0.0268 (8)	0.0155 (6)	0.0216 (7)	0.0002 (6)	0.0181 (7)	−0.0038 (5)
C2	0.0240 (8)	0.0133 (6)	0.0241 (7)	0.0044 (5)	0.0177 (7)	0.0011 (5)
C9	0.0169 (7)	0.0136 (6)	0.0144 (6)	0.0041 (5)	0.0085 (6)	0.0037 (5)
C13	0.0166 (7)	0.0188 (7)	0.0181 (7)	−0.0049 (5)	0.0105 (6)	−0.0053 (5)
C7	0.0167 (7)	0.0179 (6)	0.0103 (6)	0.0007 (5)	0.0078 (6)	0.0006 (5)
C1	0.0178 (7)	0.0133 (6)	0.0159 (6)	0.0037 (5)	0.0111 (6)	0.0029 (5)
C8	0.0177 (7)	0.0183 (7)	0.0123 (6)	0.0033 (5)	0.0080 (6)	0.0047 (5)
Si1	0.01073 (18)	0.00884 (15)	0.00999 (16)	0.00026 (13)	0.00624 (15)	0.00056 (12)
F4	0.0190 (5)	0.0192 (4)	0.0132 (4)	0.0048 (3)	0.0070 (4)	0.0019 (3)
F2	0.0299 (5)	0.0312 (5)	0.0166 (4)	−0.0035 (4)	0.0171 (4)	−0.0048 (4)
F6	0.0171 (4)	0.0126 (4)	0.0194 (4)	−0.0030 (3)	0.0065 (4)	0.0002 (3)
F3	0.0205 (5)	0.0198 (4)	0.0144 (4)	−0.0078 (3)	0.0103 (4)	−0.0032 (3)
F5	0.0360 (6)	0.0175 (4)	0.0397 (6)	−0.0025 (4)	0.0331 (5)	−0.0043 (4)
F1	0.0198 (5)	0.0147 (4)	0.0271 (5)	0.0010 (4)	0.0063 (4)	0.0065 (4)
O2	0.0311 (7)	0.0166 (6)	0.0643 (10)	0.0028 (5)	0.0371 (8)	0.0061 (6)
O3	0.0239 (7)	0.0252 (6)	0.0312 (7)	−0.0023 (5)	0.0204 (6)	−0.0025 (5)
O4	0.0253 (7)	0.0281 (6)	0.0154 (6)	−0.0004 (5)	0.0116 (6)	0.0012 (5)
O5	0.0242 (7)	0.0280 (6)	0.0323 (7)	0.0018 (5)	0.0207 (7)	0.0037 (5)

### Aquabis(2,2'-bipyridine-κ2N,N')copper(II) hexafluoridosilicate tetrahydrate (I) Geometric parameters (Å, º)

**Table:**

Cu1—O1	2.1112 (11)	C20—C19	1.3831 (19)
Cu1—N1	2.0419 (12)	C14—H14	0.9500
Cu1—N3	2.0849 (12)	C14—C13	1.388 (2)
Cu1—N4	1.9760 (11)	C19—H19	0.9500
Cu1—N2	1.9730 (11)	C10—H10	0.9500
O1—H1A	0.76 (3)	C10—C9	1.3841 (19)
O1—H1B	0.78 (2)	C3—H3	0.9500
N1—C5	1.3527 (17)	C3—C2	1.383 (2)
N1—C1	1.3401 (17)	C2—H2	0.9500
N3—C15	1.3528 (17)	C2—C1	1.383 (2)
N3—C11	1.3368 (17)	C9—H9	0.9500
N4—C16	1.3525 (18)	C9—C8	1.383 (2)
N4—C20	1.3392 (18)	C13—H13	0.9500
N2—C6	1.3510 (17)	C7—H7	0.9500
N2—C10	1.3379 (18)	C7—C8	1.385 (2)
C6—C5	1.4749 (18)	C1—H1	0.9500
C6—C7	1.3852 (18)	C8—H8	0.9500
C16—C15	1.4761 (19)	Si1—F4	1.6918 (9)
C16—C17	1.3845 (19)	Si1—F2	1.6708 (10)
C15—C14	1.3860 (19)	Si1—F6	1.6886 (9)
C17—H17	0.9500	Si1—F3	1.6947 (9)
C17—C18	1.386 (2)	Si1—F5	1.6695 (10)
C4—H4	0.9500	Si1—F1	1.6677 (10)
C4—C5	1.3883 (19)	O2—H2A	0.75 (2)
C4—C3	1.387 (2)	O2—H2B	0.79 (3)
C11—H11	0.9500	O3—H3A	0.77 (3)
C11—C12	1.380 (2)	O3—H3B	0.73 (3)
C18—H18	0.9500	O4—H4A	0.72 (2)
C18—C19	1.376 (2)	O4—H4B	0.77 (3)
C12—H12	0.9500	O5—H5A	0.69 (2)
C12—C13	1.382 (2)	O5—H5B	0.81 (3)
C20—H20	0.9500		
			
N1—Cu1—O1	127.84 (5)	N4—C20—H20	118.9
N1—Cu1—N3	132.07 (5)	N4—C20—C19	122.15 (14)
N3—Cu1—O1	100.04 (5)	C19—C20—H20	118.9
N4—Cu1—O1	92.43 (5)	C15—C14—H14	120.4
N4—Cu1—N1	97.75 (5)	C15—C14—C13	119.29 (13)
N4—Cu1—N3	80.40 (5)	C13—C14—H14	120.4
N2—Cu1—O1	88.34 (5)	C18—C19—C20	118.74 (13)
N2—Cu1—N1	80.70 (5)	C18—C19—H19	120.6
N2—Cu1—N3	100.81 (5)	C20—C19—H19	120.6
N2—Cu1—N4	178.43 (5)	N2—C10—H10	119.0
Cu1—O1—H1A	118.2 (18)	N2—C10—C9	122.06 (13)
Cu1—O1—H1B	114.6 (17)	C9—C10—H10	119.0
H1A—O1—H1B	109 (2)	C4—C3—H3	120.4
C5—N1—Cu1	113.79 (9)	C2—C3—C4	119.18 (13)
C1—N1—Cu1	127.88 (10)	C2—C3—H3	120.4
C1—N1—C5	118.32 (12)	C3—C2—H2	120.6
C15—N3—Cu1	112.90 (9)	C1—C2—C3	118.80 (14)
C11—N3—Cu1	128.87 (10)	C1—C2—H2	120.6
C11—N3—C15	118.23 (12)	C10—C9—H9	120.6
C16—N4—Cu1	116.39 (9)	C8—C9—C10	118.78 (13)
C20—N4—Cu1	124.34 (10)	C8—C9—H9	120.6
C20—N4—C16	119.23 (12)	C12—C13—C14	118.78 (14)
C6—N2—Cu1	116.33 (9)	C12—C13—H13	120.6
C10—N2—Cu1	124.23 (9)	C14—C13—H13	120.6
C10—N2—C6	119.40 (12)	C6—C7—H7	120.4
N2—C6—C5	114.45 (11)	C6—C7—C8	119.10 (13)
N2—C6—C7	121.29 (12)	C8—C7—H7	120.4
C7—C6—C5	124.26 (12)	N1—C1—C2	122.79 (13)
N4—C16—C15	115.22 (12)	N1—C1—H1	118.6
N4—C16—C17	121.31 (13)	C2—C1—H1	118.6
C17—C16—C15	123.42 (13)	C9—C8—C7	119.32 (13)
N3—C15—C16	114.92 (12)	C9—C8—H8	120.3
N3—C15—C14	121.76 (13)	C7—C8—H8	120.3
C14—C15—C16	123.29 (12)	F4—Si1—F3	90.21 (5)
C16—C17—H17	120.5	F2—Si1—F4	89.52 (5)
C16—C17—C18	118.93 (14)	F2—Si1—F6	90.16 (5)
C18—C17—H17	120.5	F2—Si1—F3	89.51 (5)
C5—C4—H4	120.6	F6—Si1—F4	89.03 (5)
C3—C4—H4	120.6	F6—Si1—F3	179.18 (5)
C3—C4—C5	118.83 (13)	F5—Si1—F4	89.80 (5)
N1—C5—C6	114.73 (11)	F5—Si1—F2	178.71 (6)
N1—C5—C4	122.06 (13)	F5—Si1—F6	90.92 (5)
C4—C5—C6	123.21 (12)	F5—Si1—F3	89.40 (5)
N3—C11—H11	118.4	F1—Si1—F4	179.43 (6)
N3—C11—C12	123.13 (13)	F1—Si1—F2	89.97 (6)
C12—C11—H11	118.4	F1—Si1—F6	90.70 (5)
C17—C18—H18	120.2	F1—Si1—F3	90.05 (5)
C19—C18—C17	119.62 (13)	F1—Si1—F5	90.71 (6)
C19—C18—H18	120.2	H2A—O2—H2B	106 (2)
C11—C12—H12	120.6	H3A—O3—H3B	103 (3)
C11—C12—C13	118.79 (13)	H4A—O4—H4B	105 (2)
C13—C12—H12	120.6	H5A—O5—H5B	107 (2)

### Aquabis(2,2'-bipyridine-κ2N,N')copper(II) hexafluoridosilicate tetrahydrate (I) Hydrogen-bond geometry (Å, º)

**Table:**

*D*—H···*A*	*D*—H	H···*A*	*D*···*A*	*D*—H···*A*
O1—H1*A*···F3	0.76 (3)	1.91 (3)	2.6616 (14)	177 (3)
O1—H1*B*···F6^i^	0.78 (2)	1.93 (2)	2.7053 (14)	170 (2)
O2—H2*A*···F1	0.75 (2)	1.94 (2)	2.6677 (17)	164 (2)
O2—H2*B*···F4^i^	0.79 (3)	2.00 (3)	2.7807 (17)	167 (2)
O3—H3*A*···F5^ii^	0.77 (3)	1.99 (3)	2.7607 (18)	177 (3)
O3—H3*B*···O5^iii^	0.73 (3)	2.06 (3)	2.779 (2)	171 (3)
O4—H4*A*···O3	0.72 (2)	2.05 (2)	2.749 (2)	162 (2)
O4—H4*B*···F4	0.77 (3)	1.98 (3)	2.7462 (16)	170 (2)
O5—H5*A*···O4	0.69 (2)	2.13 (2)	2.779 (2)	158 (2)
O5—H5*B*···O2^iv^	0.81 (3)	1.99 (3)	2.786 (2)	169 (2)

Symmetry codes: (i) −*x*+1/2, *y*+1/2, −*z*+1/2; (ii) −*x*+1/2, −*y*+1/2, −*z*+1; (iii) −*x*+1, *y*, −*z*+3/2; (iv) *x*+1/2, −*y*+1/2, *z*+1/2.

### Bis(2,2'-bipyridine-3κ2N,N')-di-µ-fluorido-1:3κ2F:F;2:3κ2F:F-decafluorido-1κ5F,2κ5F-copper(II)ditantalum(V) (II) Crystal data

**Table:**

[CuTa_2_F_12_(C_10_H_8_N_2_)_2_]	*Z* = 4
*M**_r_* = 965.81	*F*(000) = 1788
Triclinic, *P*1	*D*_x_ = 2.543 Mg m^−^^3^
*a* = 9.5465 (1) Å	Mo *K*α radiation, λ = 0.71073 Å
*b* = 10.5102 (1) Å	Cell parameters from 52090 reflections
*c* = 25.9853 (4) Å	θ = 2.1–33.9°
α = 96.723 (1)°	µ = 9.60 mm^−^^1^
β = 100.256 (1)°	*T* = 100 K
γ = 96.672 (1)°	Plate, blue
*V* = 2522.78 (5) Å^3^	0.52 × 0.32 × 0.22 mm

### Bis(2,2'-bipyridine-3κ2N,N')-di-µ-fluorido-1:3κ2F:F;2:3κ2F:F-decafluorido-1κ5F,2κ5F-copper(II)ditantalum(V) (II) Data collection

**Table:**

Rigaku Oxford Diffraction XtaLAB Synergy, Single source at offset/far, HyPix diffractometer	18263 independent reflections
Radiation source: micro-focus sealed X-ray tube, PhotonJet (Mo) X-ray Source	15170 reflections with *I* > 2σ(*I*)
Mirror monochromator	*R*_int_ = 0.060
Detector resolution: 10.0000 pixels mm^-1^	θ_max_ = 33.9°, θ_min_ = 2.0°
ω scans	*h* = −14→13
Absorption correction: gaussian Crysalispro (Rigaku OD, 2020)	*k* = −16→16
*T*_min_ = 0.035, *T*_max_ = 0.414	*l* = −40→39
93570 measured reflections	

### Bis(2,2'-bipyridine-3κ2N,N')-di-µ-fluorido-1:3κ2F:F;2:3κ2F:F-decafluorido-1κ5F,2κ5F-copper(II)ditantalum(V) (II) Refinement

**Table:**

Refinement on *F*^2^	Hydrogen site location: inferred from neighbouring sites
Least-squares matrix: full	H-atom parameters constrained
*R*[*F*^2^ > 2σ(*F*^2^)] = 0.037	*w* = 1/[σ^2^(*F*_o_^2^) + (0.0409*P*)^2^ + 5.1565*P*] where *P* = (*F*_o_^2^ + 2*F*_c_^2^)/3
*wR*(*F*^2^) = 0.088	(Δ/σ)_max_ = 0.002
*S* = 1.05	Δρ_max_ = 1.67 e Å^−^^3^
18263 reflections	Δρ_min_ = −3.53 e Å^−^^3^
704 parameters	Extinction correction: SHELXL-2018/3 (Sheldrick 2015b), Fc^*^=kFc[1+0.001xFc^2^λ^3^/sin(2θ)]^-1/4^
0 restraints	Extinction coefficient: 0.00034 (4)

### Bis(2,2'-bipyridine-3κ2N,N')-di-µ-fluorido-1:3κ2F:F;2:3κ2F:F-decafluorido-1κ5F,2κ5F-copper(II)ditantalum(V) (II) Special details

**Table:**

Geometry. All esds (except the esd in the dihedral angle between two l.s. planes) are estimated using the full covariance matrix. The cell esds are taken into account individually in the estimation of esds in distances, angles and torsion angles; correlations between esds in cell parameters are only used when they are defined by crystal symmetry. An approximate (isotropic) treatment of cell esds is used for estimating esds involving l.s. planes.

### Bis(2,2'-bipyridine-3κ2N,N')-di-µ-fluorido-1:3κ2F:F;2:3κ2F:F-decafluorido-1κ5F,2κ5F-copper(II)ditantalum(V) (II) Fractional atomic coordinates and isotropic or equivalent isotropic displacement parameters (Å^2^)

**Table:**

	*x*	*y*	*z*	*U*_iso_*/*U*_eq_	
Ta2	0.98596 (2)	1.10178 (2)	0.12906 (2)	0.01573 (4)	
Ta1	0.41338 (2)	0.45975 (2)	0.10819 (2)	0.01761 (4)	
Cu1	0.68618 (5)	0.78568 (5)	0.13192 (2)	0.01516 (9)	
F9	0.9510 (3)	1.1887 (3)	0.19295 (10)	0.0252 (5)	
F5	0.2489 (3)	0.4552 (3)	0.14002 (11)	0.0267 (6)	
F8	0.7872 (3)	1.0564 (3)	0.10038 (11)	0.0260 (6)	
F12	1.0286 (3)	1.0247 (3)	0.06509 (10)	0.0239 (5)	
F11	1.1860 (3)	1.1389 (3)	0.15719 (11)	0.0262 (6)	
F3	0.5185 (3)	0.4088 (3)	0.16953 (11)	0.0247 (5)	
F7	0.9770 (3)	0.9436 (3)	0.15757 (11)	0.0294 (6)	
F4	0.3621 (3)	0.2852 (3)	0.07704 (12)	0.0305 (6)	
F10	0.9810 (3)	1.2592 (2)	0.10011 (11)	0.0256 (6)	
F2	0.5792 (3)	0.4746 (3)	0.07763 (12)	0.0331 (6)	
F6	0.3068 (3)	0.5133 (3)	0.04785 (11)	0.0270 (6)	
F1	0.4651 (3)	0.6364 (2)	0.14024 (11)	0.0269 (6)	
N3	0.7804 (4)	0.6735 (3)	0.17963 (14)	0.0160 (6)	
N1	0.5349 (3)	0.8408 (3)	0.08062 (14)	0.0157 (6)	
N4	0.8057 (4)	0.7153 (3)	0.08444 (14)	0.0152 (6)	
N2	0.6440 (4)	0.9307 (3)	0.17981 (14)	0.0157 (6)	
C2	0.3580 (5)	0.8158 (4)	0.00134 (18)	0.0216 (8)	
H2	0.319847	0.772106	−0.033323	0.026*	
C20	0.8274 (4)	0.7603 (4)	0.04025 (17)	0.0175 (7)	
H20	0.767028	0.818420	0.025928	0.021*	
C17	0.9999 (5)	0.5935 (4)	0.08156 (18)	0.0215 (8)	
H17	1.057912	0.534030	0.096134	0.026*	
C5	0.4765 (4)	0.9423 (4)	0.10083 (16)	0.0152 (7)	
C7	0.5236 (4)	1.1185 (4)	0.17918 (17)	0.0185 (8)	
H7	0.455457	1.165969	0.161648	0.022*	
C6	0.5475 (4)	1.0002 (4)	0.15501 (16)	0.0162 (7)	
C11	0.7482 (4)	0.6497 (4)	0.22631 (17)	0.0189 (8)	
H11	0.688936	0.702667	0.242198	0.023*	
C12	0.7986 (5)	0.5507 (4)	0.25202 (17)	0.0200 (8)	
H12	0.777478	0.537798	0.285559	0.024*	
C15	0.8624 (4)	0.5978 (4)	0.15622 (16)	0.0157 (7)	
C8	0.6014 (5)	1.1659 (4)	0.22958 (17)	0.0206 (8)	
H8	0.588245	1.247440	0.246647	0.025*	
C19	0.9357 (5)	0.7250 (4)	0.01437 (17)	0.0208 (8)	
H19	0.948827	0.757597	−0.017295	0.025*	
C9	0.6985 (5)	1.0938 (4)	0.25498 (18)	0.0211 (8)	
H9	0.750625	1.123962	0.289801	0.025*	
C3	0.2968 (4)	0.9182 (4)	0.02197 (17)	0.0207 (8)	
H3	0.214765	0.944598	0.001826	0.025*	
C16	0.8910 (4)	0.6331 (4)	0.10567 (16)	0.0158 (7)	
C1	0.4762 (4)	0.7779 (4)	0.03212 (16)	0.0182 (7)	
H1	0.516609	0.705646	0.018558	0.022*	
C14	0.9133 (4)	0.4946 (4)	0.17934 (17)	0.0197 (8)	
H14	0.969857	0.440928	0.162279	0.024*	
C4	0.3562 (4)	0.9825 (4)	0.07255 (17)	0.0184 (8)	
H4	0.315036	1.052700	0.087389	0.022*	
C18	1.0233 (5)	0.6418 (4)	0.03578 (18)	0.0230 (8)	
H18	1.099428	0.617504	0.019383	0.028*	
C10	0.7175 (4)	0.9771 (4)	0.22842 (16)	0.0179 (7)	
H10	0.785232	0.928262	0.245294	0.021*	
C13	0.8803 (5)	0.4712 (4)	0.22762 (18)	0.0216 (8)	
H13	0.913687	0.400990	0.243799	0.026*	
Ta3	−0.00635 (2)	0.33576 (2)	0.38898 (2)	0.02034 (4)	
Ta4	0.63142 (2)	−0.13811 (2)	0.36861 (2)	0.02023 (4)	
Cu2	0.29152 (6)	0.10318 (5)	0.36059 (2)	0.01799 (10)	
F21	0.8047 (3)	−0.0728 (3)	0.34984 (13)	0.0329 (7)	
F13	0.0337 (3)	0.1905 (3)	0.34529 (12)	0.0312 (6)	
F23	0.5569 (3)	−0.2131 (3)	0.29738 (11)	0.0307 (6)	
F16	−0.0387 (3)	0.4811 (3)	0.43312 (13)	0.0359 (7)	
F24	0.4541 (3)	−0.2063 (4)	0.38472 (13)	0.0458 (9)	
F15	−0.1820 (3)	0.3384 (3)	0.34261 (12)	0.0359 (7)	
F19	0.5514 (3)	0.0146 (3)	0.35417 (13)	0.0383 (7)	
F22	0.7064 (3)	−0.2934 (3)	0.38108 (16)	0.0460 (9)	
F14	−0.0971 (4)	0.2243 (3)	0.42789 (14)	0.0484 (9)	
F20	0.6966 (4)	−0.0686 (4)	0.44044 (13)	0.0504 (9)	
N7	0.1943 (4)	−0.0253 (3)	0.39661 (14)	0.0170 (6)	
F17	0.0823 (4)	0.4475 (3)	0.34739 (15)	0.0440 (8)	
N5	0.3281 (4)	0.2525 (3)	0.32378 (14)	0.0182 (7)	
N6	0.4368 (4)	0.2126 (3)	0.41895 (14)	0.0181 (7)	
N8	0.2405 (4)	−0.0415 (3)	0.29996 (15)	0.0191 (7)	
F18	0.1739 (4)	0.3349 (4)	0.43259 (17)	0.0627 (12)	
C39	0.2640 (5)	−0.1563 (4)	0.21782 (18)	0.0218 (8)	
H39	0.291350	−0.154459	0.184489	0.026*	
C26	0.4974 (4)	0.3223 (4)	0.40414 (17)	0.0178 (7)	
C23	0.3995 (5)	0.4718 (4)	0.2798 (2)	0.0259 (9)	
H23	0.426235	0.545695	0.264082	0.031*	
C35	0.1492 (4)	−0.1430 (4)	0.36712 (16)	0.0168 (7)	
C33	0.0475 (5)	−0.2200 (4)	0.43722 (18)	0.0221 (8)	
H33	−0.002722	−0.287185	0.451181	0.027*	
C37	0.1677 (4)	−0.2708 (4)	0.28085 (17)	0.0199 (8)	
H37	0.129683	−0.348965	0.291495	0.024*	
C22	0.2878 (5)	0.3774 (4)	0.25324 (18)	0.0233 (8)	
H22	0.234903	0.387033	0.219656	0.028*	
C31	0.1648 (4)	−0.0038 (4)	0.44524 (17)	0.0202 (8)	
H31	0.192692	0.080082	0.465036	0.024*	
C34	0.0740 (5)	−0.2408 (4)	0.38662 (18)	0.0212 (8)	
H34	0.040589	−0.322079	0.365254	0.025*	
C27	0.6082 (5)	0.4046 (5)	0.43749 (19)	0.0254 (9)	
H27	0.649078	0.480936	0.426353	0.030*	
C32	0.0950 (5)	−0.1001 (4)	0.46741 (18)	0.0217 (8)	
H32	0.079950	−0.084253	0.502625	0.026*	
C36	0.1841 (4)	−0.1539 (4)	0.31391 (16)	0.0166 (7)	
C29	0.5970 (5)	0.2631 (5)	0.50230 (19)	0.0272 (9)	
H29	0.629406	0.241474	0.536481	0.033*	
C21	0.2552 (5)	0.2689 (4)	0.27675 (17)	0.0214 (8)	
H21	0.178536	0.204241	0.258864	0.026*	
C25	0.4332 (4)	0.3463 (4)	0.35056 (17)	0.0180 (7)	
C24	0.4716 (5)	0.4571 (4)	0.32964 (18)	0.0226 (8)	
H24	0.545932	0.522120	0.348967	0.027*	
C38	0.2078 (5)	−0.2715 (4)	0.23199 (19)	0.0232 (9)	
H38	0.196865	−0.350143	0.208612	0.028*	
C30	0.4876 (5)	0.1833 (4)	0.46724 (17)	0.0215 (8)	
H30	0.447016	0.105492	0.477449	0.026*	
C40	0.2796 (5)	−0.0435 (4)	0.25308 (17)	0.0215 (8)	
H40	0.319631	0.035248	0.243508	0.026*	
C28	0.6592 (5)	0.3750 (5)	0.4873 (2)	0.0293 (10)	
H28	0.735536	0.430406	0.510793	0.035*	

### Bis(2,2'-bipyridine-3κ2N,N')-di-µ-fluorido-1:3κ2F:F;2:3κ2F:F-decafluorido-1κ5F,2κ5F-copper(II)ditantalum(V) (II) Atomic displacement parameters (Å^2^)

**Table:**

	*U*^11^	*U*^22^	*U*^33^	*U*^12^	*U*^13^	*U*^23^
Ta2	0.01384 (7)	0.01637 (7)	0.01688 (8)	0.00172 (5)	0.00301 (6)	0.00224 (6)
Ta1	0.01627 (8)	0.01625 (8)	0.02005 (8)	0.00161 (6)	0.00173 (6)	0.00487 (6)
Cu1	0.0163 (2)	0.0154 (2)	0.0156 (2)	0.00803 (17)	0.00351 (17)	0.00329 (17)
F9	0.0252 (13)	0.0316 (14)	0.0196 (13)	0.0051 (11)	0.0082 (10)	0.0003 (10)
F5	0.0206 (12)	0.0359 (15)	0.0268 (14)	0.0074 (11)	0.0059 (10)	0.0120 (12)
F8	0.0158 (11)	0.0372 (15)	0.0237 (13)	−0.0007 (10)	0.0022 (10)	0.0056 (11)
F12	0.0263 (13)	0.0245 (13)	0.0211 (13)	0.0048 (10)	0.0066 (10)	−0.0008 (10)
F11	0.0153 (11)	0.0350 (15)	0.0263 (14)	0.0025 (10)	0.0027 (10)	−0.0012 (11)
F3	0.0234 (12)	0.0226 (12)	0.0267 (14)	0.0041 (10)	−0.0023 (10)	0.0076 (10)
F7	0.0364 (15)	0.0221 (13)	0.0290 (15)	0.0037 (11)	0.0015 (12)	0.0091 (11)
F4	0.0407 (16)	0.0173 (12)	0.0287 (15)	0.0020 (11)	−0.0023 (12)	0.0000 (11)
F10	0.0352 (15)	0.0184 (12)	0.0280 (14)	0.0070 (10)	0.0140 (11)	0.0069 (10)
F2	0.0247 (14)	0.0439 (17)	0.0319 (16)	0.0030 (12)	0.0093 (12)	0.0062 (13)
F6	0.0278 (13)	0.0303 (14)	0.0227 (13)	0.0026 (11)	0.0000 (11)	0.0117 (11)
F1	0.0296 (14)	0.0183 (12)	0.0300 (15)	0.0009 (10)	0.0004 (11)	0.0029 (10)
N3	0.0158 (15)	0.0143 (14)	0.0187 (16)	0.0053 (12)	0.0041 (12)	0.0016 (12)
N1	0.0146 (14)	0.0137 (14)	0.0202 (17)	0.0051 (11)	0.0038 (12)	0.0038 (12)
N4	0.0160 (15)	0.0129 (14)	0.0174 (16)	0.0022 (11)	0.0033 (12)	0.0038 (12)
N2	0.0160 (15)	0.0150 (14)	0.0175 (16)	0.0050 (12)	0.0046 (12)	0.0029 (12)
C2	0.0221 (19)	0.0216 (19)	0.020 (2)	0.0054 (15)	−0.0001 (16)	0.0030 (16)
C20	0.0166 (17)	0.0168 (17)	0.0206 (19)	0.0036 (14)	0.0042 (15)	0.0069 (14)
C17	0.0194 (19)	0.022 (2)	0.024 (2)	0.0082 (15)	0.0056 (16)	0.0004 (16)
C5	0.0154 (16)	0.0151 (16)	0.0179 (18)	0.0046 (13)	0.0060 (14)	0.0066 (14)
C7	0.0164 (17)	0.0166 (17)	0.024 (2)	0.0054 (14)	0.0058 (15)	0.0023 (15)
C6	0.0149 (17)	0.0159 (17)	0.0205 (19)	0.0054 (13)	0.0062 (14)	0.0061 (14)
C11	0.0187 (18)	0.0192 (18)	0.0190 (19)	0.0046 (14)	0.0036 (15)	0.0022 (15)
C12	0.0231 (19)	0.0213 (19)	0.0171 (19)	0.0032 (15)	0.0043 (15)	0.0082 (15)
C15	0.0139 (16)	0.0158 (17)	0.0175 (18)	0.0043 (13)	0.0022 (14)	0.0016 (14)
C8	0.0221 (19)	0.0179 (18)	0.022 (2)	0.0032 (15)	0.0071 (16)	−0.0005 (15)
C19	0.0218 (19)	0.0219 (19)	0.020 (2)	0.0021 (15)	0.0085 (16)	0.0030 (15)
C9	0.0214 (19)	0.0197 (19)	0.021 (2)	0.0008 (15)	0.0043 (16)	−0.0010 (15)
C3	0.0170 (18)	0.024 (2)	0.021 (2)	0.0066 (15)	0.0005 (15)	0.0056 (16)
C16	0.0163 (17)	0.0135 (16)	0.0176 (18)	0.0029 (13)	0.0032 (14)	0.0013 (13)
C1	0.0207 (18)	0.0179 (18)	0.0177 (19)	0.0067 (15)	0.0046 (15)	0.0032 (14)
C14	0.0188 (18)	0.0176 (18)	0.023 (2)	0.0048 (14)	0.0033 (15)	0.0016 (15)
C4	0.0164 (17)	0.0169 (17)	0.024 (2)	0.0069 (14)	0.0039 (15)	0.0047 (15)
C18	0.021 (2)	0.025 (2)	0.025 (2)	0.0069 (16)	0.0092 (17)	0.0003 (17)
C10	0.0192 (18)	0.0190 (18)	0.0163 (18)	0.0045 (14)	0.0037 (14)	0.0033 (14)
C13	0.023 (2)	0.0180 (18)	0.023 (2)	0.0063 (15)	−0.0007 (16)	0.0056 (15)
Ta3	0.02110 (8)	0.01596 (8)	0.02421 (9)	0.00380 (6)	0.00382 (6)	0.00350 (6)
Ta4	0.01788 (8)	0.02380 (9)	0.01855 (9)	0.00452 (6)	0.00136 (6)	0.00287 (6)
Cu2	0.0231 (2)	0.0138 (2)	0.0162 (2)	−0.00050 (18)	0.00276 (19)	0.00333 (17)
F21	0.0188 (13)	0.0342 (15)	0.0464 (18)	0.0008 (11)	0.0027 (12)	0.0167 (14)
F13	0.0352 (15)	0.0216 (13)	0.0429 (18)	0.0083 (11)	0.0209 (13)	0.0048 (12)
F23	0.0284 (14)	0.0391 (16)	0.0215 (14)	0.0037 (12)	0.0046 (11)	−0.0073 (12)
F16	0.0457 (18)	0.0243 (14)	0.0370 (17)	0.0067 (13)	0.0116 (14)	−0.0048 (12)
F24	0.0234 (14)	0.084 (3)	0.0337 (18)	0.0028 (16)	0.0096 (13)	0.0226 (17)
F15	0.0379 (16)	0.0344 (16)	0.0317 (16)	0.0131 (13)	−0.0044 (13)	−0.0014 (13)
F19	0.0375 (16)	0.0310 (16)	0.0461 (19)	0.0211 (13)	0.0008 (14)	−0.0011 (14)
F22	0.0273 (15)	0.0263 (15)	0.078 (3)	0.0024 (12)	−0.0137 (16)	0.0199 (16)
F14	0.078 (3)	0.0344 (17)	0.049 (2)	0.0158 (17)	0.0395 (19)	0.0197 (15)
F20	0.065 (2)	0.057 (2)	0.0195 (15)	−0.0035 (18)	−0.0066 (15)	−0.0008 (14)
N7	0.0184 (15)	0.0156 (15)	0.0169 (16)	0.0031 (12)	0.0034 (12)	0.0013 (12)
F17	0.057 (2)	0.0232 (14)	0.059 (2)	−0.0018 (14)	0.0350 (18)	0.0055 (14)
N5	0.0195 (16)	0.0171 (15)	0.0180 (16)	0.0004 (12)	0.0040 (13)	0.0043 (12)
N6	0.0187 (16)	0.0181 (16)	0.0180 (16)	0.0049 (13)	0.0036 (13)	0.0018 (13)
N8	0.0195 (16)	0.0139 (15)	0.0237 (18)	0.0016 (12)	0.0044 (13)	0.0026 (13)
F18	0.041 (2)	0.071 (3)	0.064 (3)	0.0237 (19)	−0.0188 (18)	−0.012 (2)
C39	0.0202 (19)	0.025 (2)	0.019 (2)	0.0033 (16)	0.0034 (15)	−0.0007 (16)
C26	0.0147 (17)	0.0155 (17)	0.023 (2)	0.0020 (13)	0.0037 (15)	0.0021 (14)
C23	0.030 (2)	0.020 (2)	0.031 (2)	0.0057 (17)	0.0095 (19)	0.0116 (17)
C35	0.0135 (16)	0.0165 (17)	0.0200 (19)	0.0014 (13)	0.0018 (14)	0.0037 (14)
C33	0.0183 (18)	0.022 (2)	0.028 (2)	0.0023 (15)	0.0066 (16)	0.0101 (17)
C37	0.0213 (19)	0.0141 (17)	0.023 (2)	0.0011 (14)	0.0027 (16)	0.0014 (15)
C22	0.026 (2)	0.024 (2)	0.022 (2)	0.0052 (17)	0.0053 (17)	0.0082 (16)
C31	0.0211 (19)	0.0186 (18)	0.022 (2)	0.0045 (15)	0.0045 (16)	0.0049 (15)
C34	0.0204 (19)	0.0154 (18)	0.028 (2)	0.0007 (15)	0.0048 (16)	0.0053 (16)
C27	0.020 (2)	0.027 (2)	0.027 (2)	−0.0026 (16)	0.0034 (17)	0.0016 (18)
C32	0.0207 (19)	0.023 (2)	0.025 (2)	0.0065 (16)	0.0071 (16)	0.0086 (16)
C36	0.0125 (16)	0.0164 (17)	0.0197 (19)	0.0006 (13)	0.0008 (14)	0.0032 (14)
C29	0.020 (2)	0.039 (3)	0.022 (2)	0.0058 (18)	0.0002 (17)	0.0043 (19)
C21	0.0223 (19)	0.0221 (19)	0.020 (2)	0.0009 (16)	0.0050 (16)	0.0050 (16)
C25	0.0167 (17)	0.0158 (17)	0.022 (2)	0.0039 (14)	0.0047 (15)	0.0036 (15)
C24	0.024 (2)	0.0172 (18)	0.026 (2)	−0.0018 (15)	0.0049 (17)	0.0045 (16)
C38	0.0195 (19)	0.021 (2)	0.027 (2)	0.0038 (15)	0.0021 (16)	−0.0019 (16)
C30	0.0205 (19)	0.025 (2)	0.019 (2)	0.0028 (16)	0.0031 (15)	0.0041 (16)
C40	0.026 (2)	0.0195 (19)	0.019 (2)	0.0031 (16)	0.0027 (16)	0.0043 (15)
C28	0.022 (2)	0.036 (3)	0.026 (2)	−0.0040 (18)	0.0003 (18)	0.0021 (19)

### Bis(2,2'-bipyridine-3κ2N,N')-di-µ-fluorido-1:3κ2F:F;2:3κ2F:F-decafluorido-1κ5F,2κ5F-copper(II)ditantalum(V) (II) Geometric parameters (Å, º)

**Table:**

Ta2—F9	1.902 (3)	Ta3—F13	1.910 (3)
Ta2—F8	1.893 (2)	Ta3—F16	1.889 (3)
Ta2—F12	1.894 (3)	Ta3—F15	1.887 (3)
Ta2—F11	1.896 (2)	Ta3—F14	1.870 (3)
Ta2—F7	1.897 (3)	Ta3—F17	1.912 (3)
Ta2—F10	1.898 (3)	Ta3—F18	1.886 (3)
Ta1—F5	1.899 (3)	Ta4—F21	1.883 (3)
Ta1—F3	1.894 (3)	Ta4—F23	1.901 (3)
Ta1—F4	1.888 (3)	Ta4—F24	1.896 (3)
Ta1—F2	1.892 (3)	Ta4—F19	1.903 (3)
Ta1—F6	1.893 (3)	Ta4—F22	1.895 (3)
Ta1—F1	1.913 (3)	Ta4—F20	1.889 (3)
Cu1—F7	2.987 (3)	Cu2—F13	2.706 (3)
Cu1—F1	2.537 (3)	Cu2—F19	2.775 (3)
Cu1—N3	1.982 (3)	Cu2—N7	1.968 (3)
Cu1—N1	1.972 (3)	Cu2—N5	1.960 (3)
Cu1—N4	1.964 (3)	Cu2—N6	2.005 (4)
Cu1—N2	1.977 (3)	Cu2—N8	2.006 (4)
N3—C11	1.347 (5)	N7—C35	1.356 (5)
N3—C15	1.348 (5)	N7—C31	1.342 (5)
N1—C5	1.354 (5)	N5—C21	1.335 (6)
N1—C1	1.344 (5)	N5—C25	1.351 (5)
N4—C20	1.332 (5)	N6—C26	1.356 (5)
N4—C16	1.353 (5)	N6—C30	1.346 (6)
N2—C6	1.362 (5)	N8—C36	1.356 (5)
N2—C10	1.337 (5)	N8—C40	1.335 (6)
C2—H2	0.9500	C39—H39	0.9500
C2—C3	1.382 (6)	C39—C38	1.383 (6)
C2—C1	1.390 (6)	C39—C40	1.386 (6)
C20—H20	0.9500	C26—C27	1.379 (6)
C20—C19	1.392 (6)	C26—C25	1.481 (6)
C17—H17	0.9500	C23—H23	0.9500
C17—C16	1.385 (6)	C23—C22	1.389 (7)
C17—C18	1.390 (6)	C23—C24	1.388 (7)
C5—C6	1.475 (6)	C35—C34	1.382 (6)
C5—C4	1.387 (5)	C35—C36	1.474 (6)
C7—H7	0.9500	C33—H33	0.9500
C7—C6	1.388 (5)	C33—C34	1.380 (6)
C7—C8	1.389 (6)	C33—C32	1.385 (6)
C11—H11	0.9500	C37—H37	0.9500
C11—C12	1.388 (6)	C37—C36	1.390 (6)
C12—H12	0.9500	C37—C38	1.389 (6)
C12—C13	1.383 (6)	C22—H22	0.9500
C15—C16	1.468 (6)	C22—C21	1.385 (6)
C15—C14	1.395 (6)	C31—H31	0.9500
C8—H8	0.9500	C31—C32	1.385 (6)
C8—C9	1.390 (6)	C34—H34	0.9500
C19—H19	0.9500	C27—H27	0.9500
C19—C18	1.375 (6)	C27—C28	1.384 (7)
C9—H9	0.9500	C32—H32	0.9500
C9—C10	1.382 (6)	C29—H29	0.9500
C3—H3	0.9500	C29—C30	1.380 (6)
C3—C4	1.394 (6)	C29—C28	1.383 (7)
C1—H1	0.9500	C21—H21	0.9500
C14—H14	0.9500	C25—C24	1.381 (6)
C14—C13	1.389 (6)	C24—H24	0.9500
C4—H4	0.9500	C38—H38	0.9500
C18—H18	0.9500	C30—H30	0.9500
C10—H10	0.9500	C40—H40	0.9500
C13—H13	0.9500	C28—H28	0.9500
			
F8—Ta2—F9	92.66 (12)	F13—Ta3—F17	89.01 (13)
F8—Ta2—F12	89.58 (12)	F16—Ta3—F13	177.92 (14)
F8—Ta2—F11	177.33 (12)	F16—Ta3—F17	90.17 (14)
F8—Ta2—F7	87.35 (12)	F15—Ta3—F13	91.30 (13)
F8—Ta2—F10	88.94 (12)	F15—Ta3—F16	90.58 (14)
F12—Ta2—F9	176.38 (11)	F15—Ta3—F17	87.30 (16)
F12—Ta2—F11	88.98 (12)	F14—Ta3—F13	90.20 (13)
F12—Ta2—F7	92.89 (12)	F14—Ta3—F16	90.66 (14)
F12—Ta2—F10	88.43 (11)	F14—Ta3—F15	91.22 (17)
F11—Ta2—F9	88.88 (11)	F14—Ta3—F17	178.31 (17)
F11—Ta2—F7	90.47 (12)	F14—Ta3—F18	91.3 (2)
F11—Ta2—F10	93.28 (12)	F18—Ta3—F13	87.43 (16)
F7—Ta2—F9	90.05 (12)	F18—Ta3—F16	90.66 (16)
F7—Ta2—F10	176.05 (12)	F18—Ta3—F15	177.17 (18)
F10—Ta2—F9	88.78 (12)	F18—Ta3—F17	90.2 (2)
F5—Ta1—F1	87.61 (12)	F21—Ta4—F23	90.32 (13)
F3—Ta1—F5	89.44 (12)	F21—Ta4—F24	177.79 (14)
F3—Ta1—F1	89.06 (11)	F21—Ta4—F19	90.96 (14)
F4—Ta1—F5	92.10 (13)	F21—Ta4—F22	89.84 (14)
F4—Ta1—F3	90.64 (12)	F21—Ta4—F20	92.80 (16)
F4—Ta1—F2	91.12 (14)	F23—Ta4—F19	89.22 (14)
F4—Ta1—F6	90.26 (12)	F24—Ta4—F23	87.48 (14)
F4—Ta1—F1	179.59 (13)	F24—Ta4—F19	88.86 (16)
F2—Ta1—F5	176.73 (13)	F22—Ta4—F23	89.03 (15)
F2—Ta1—F3	91.08 (13)	F22—Ta4—F24	90.27 (16)
F2—Ta1—F6	89.72 (13)	F22—Ta4—F19	178.08 (14)
F2—Ta1—F1	89.17 (13)	F20—Ta4—F23	176.84 (15)
F6—Ta1—F5	89.71 (12)	F20—Ta4—F24	89.40 (16)
F6—Ta1—F3	178.79 (12)	F20—Ta4—F19	91.22 (16)
F6—Ta1—F1	90.04 (12)	F20—Ta4—F22	90.48 (17)
F1—Cu1—F7	161.46 (9)	F13—Cu2—F19	168.21 (10)
N3—Cu1—F7	83.17 (11)	N7—Cu2—F13	83.91 (12)
N3—Cu1—F1	81.03 (12)	N7—Cu2—F19	103.51 (12)
N1—Cu1—F7	118.40 (11)	N7—Cu2—N6	103.87 (14)
N1—Cu1—F1	78.18 (12)	N7—Cu2—N8	82.29 (14)
N1—Cu1—N3	158.21 (14)	N5—Cu2—F13	77.43 (12)
N1—Cu1—N2	83.13 (14)	N5—Cu2—F19	95.18 (13)
N4—Cu1—F7	70.99 (11)	N5—Cu2—N7	161.27 (15)
N4—Cu1—F1	116.26 (12)	N5—Cu2—N6	82.25 (15)
N4—Cu1—N3	82.84 (14)	N5—Cu2—N8	101.20 (15)
N4—Cu1—N1	100.59 (14)	N6—Cu2—F13	113.10 (12)
N4—Cu1—N2	150.27 (14)	N6—Cu2—F19	74.40 (12)
N2—Cu1—F7	81.24 (11)	N6—Cu2—N8	150.51 (14)
N2—Cu1—F1	93.43 (12)	N8—Cu2—F13	96.12 (12)
N2—Cu1—N3	104.65 (14)	N8—Cu2—F19	76.12 (12)
Ta2—F7—Cu1	114.17 (12)	Ta3—F13—Cu2	122.63 (14)
Ta1—F1—Cu1	125.65 (14)	Ta4—F19—Cu2	135.07 (17)
C11—N3—Cu1	126.4 (3)	C35—N7—Cu2	114.6 (3)
C11—N3—C15	119.5 (3)	C31—N7—Cu2	125.9 (3)
C15—N3—Cu1	112.8 (3)	C31—N7—C35	119.4 (4)
C5—N1—Cu1	113.7 (3)	C21—N5—Cu2	125.5 (3)
C1—N1—Cu1	126.0 (3)	C21—N5—C25	119.5 (4)
C1—N1—C5	119.8 (3)	C25—N5—Cu2	115.0 (3)
C20—N4—Cu1	125.2 (3)	C26—N6—Cu2	113.1 (3)
C20—N4—C16	119.9 (3)	C30—N6—Cu2	127.7 (3)
C16—N4—Cu1	113.6 (3)	C30—N6—C26	118.8 (4)
C6—N2—Cu1	112.9 (3)	C36—N8—Cu2	112.8 (3)
C10—N2—Cu1	126.5 (3)	C40—N8—Cu2	127.0 (3)
C10—N2—C6	119.5 (3)	C40—N8—C36	118.9 (4)
C3—C2—H2	120.5	C38—C39—H39	120.6
C3—C2—C1	118.9 (4)	C38—C39—C40	118.9 (4)
C1—C2—H2	120.5	C40—C39—H39	120.6
N4—C20—H20	118.9	N6—C26—C27	121.6 (4)
N4—C20—C19	122.2 (4)	N6—C26—C25	114.7 (3)
C19—C20—H20	118.9	C27—C26—C25	123.7 (4)
C16—C17—H17	120.4	C22—C23—H23	120.3
C16—C17—C18	119.3 (4)	C24—C23—H23	120.3
C18—C17—H17	120.4	C24—C23—C22	119.4 (4)
N1—C5—C6	114.6 (3)	N7—C35—C34	120.8 (4)
N1—C5—C4	121.3 (4)	N7—C35—C36	114.5 (3)
C4—C5—C6	124.1 (4)	C34—C35—C36	124.7 (4)
C6—C7—H7	120.7	C34—C33—H33	120.4
C6—C7—C8	118.5 (4)	C34—C33—C32	119.2 (4)
C8—C7—H7	120.7	C32—C33—H33	120.4
N2—C6—C5	114.9 (3)	C36—C37—H37	120.5
N2—C6—C7	121.4 (4)	C38—C37—H37	120.5
C7—C6—C5	123.7 (4)	C38—C37—C36	118.9 (4)
N3—C11—H11	118.9	C23—C22—H22	120.7
N3—C11—C12	122.3 (4)	C21—C22—C23	118.6 (4)
C12—C11—H11	118.9	C21—C22—H22	120.7
C11—C12—H12	120.8	N7—C31—H31	119.0
C13—C12—C11	118.4 (4)	N7—C31—C32	121.9 (4)
C13—C12—H12	120.8	C32—C31—H31	119.0
N3—C15—C16	114.7 (3)	C35—C34—H34	120.1
N3—C15—C14	121.0 (4)	C33—C34—C35	119.8 (4)
C14—C15—C16	124.3 (4)	C33—C34—H34	120.1
C7—C8—H8	120.1	C26—C27—H27	120.3
C7—C8—C9	119.9 (4)	C26—C27—C28	119.5 (4)
C9—C8—H8	120.1	C28—C27—H27	120.3
C20—C19—H19	120.8	C33—C32—H32	120.6
C18—C19—C20	118.4 (4)	C31—C32—C33	118.7 (4)
C18—C19—H19	120.8	C31—C32—H32	120.6
C8—C9—H9	120.8	N8—C36—C35	115.0 (3)
C10—C9—C8	118.5 (4)	N8—C36—C37	121.6 (4)
C10—C9—H9	120.8	C37—C36—C35	123.3 (4)
C2—C3—H3	120.2	C30—C29—H29	120.2
C2—C3—C4	119.5 (4)	C30—C29—C28	119.5 (4)
C4—C3—H3	120.2	C28—C29—H29	120.2
N4—C16—C17	120.7 (4)	N5—C21—C22	122.0 (4)
N4—C16—C15	114.5 (3)	N5—C21—H21	119.0
C17—C16—C15	124.7 (4)	C22—C21—H21	119.0
N1—C1—C2	121.6 (4)	N5—C25—C26	114.5 (3)
N1—C1—H1	119.2	N5—C25—C24	121.7 (4)
C2—C1—H1	119.2	C24—C25—C26	123.8 (4)
C15—C14—H14	120.4	C23—C24—H24	120.6
C13—C14—C15	119.1 (4)	C25—C24—C23	118.7 (4)
C13—C14—H14	120.4	C25—C24—H24	120.6
C5—C4—C3	118.9 (4)	C39—C38—C37	119.2 (4)
C5—C4—H4	120.6	C39—C38—H38	120.4
C3—C4—H4	120.6	C37—C38—H38	120.4
C17—C18—H18	120.2	N6—C30—C29	121.8 (4)
C19—C18—C17	119.6 (4)	N6—C30—H30	119.1
C19—C18—H18	120.2	C29—C30—H30	119.1
N2—C10—C9	122.3 (4)	N8—C40—C39	122.5 (4)
N2—C10—H10	118.9	N8—C40—H40	118.8
C9—C10—H10	118.9	C39—C40—H40	118.8
C12—C13—C14	119.7 (4)	C27—C28—H28	120.6
C12—C13—H13	120.2	C29—C28—C27	118.8 (4)
C14—C13—H13	120.2	C29—C28—H28	120.6

### Tris(2,2'-bipyridine-κ2N,N')copper(II) bis[hexafluoridotantalate(V)] (III) Crystal data

**Table:**

[Cu(C_10_H_8_N_2_)_3_][TaF_6_]_2_	*D*_x_ = 2.220 Mg m^−^^3^
*M**_r_* = 1121.99	Mo *K*α radiation, λ = 0.71073 Å
Trigonal, *P*3_2_	Cell parameters from 9702 reflections
*a* = 10.5172 (10) Å	θ = 2.2–32.5°
*c* = 26.288 (2) Å	µ = 7.23 mm^−^^1^
*V* = 2518.2 (5) Å^3^	*T* = 100 K
*Z* = 3	Block, blue
*F*(000) = 1587	0.22 × 0.16 × 0.12 mm

### Tris(2,2'-bipyridine-κ2N,N')copper(II) bis[hexafluoridotantalate(V)] (III) Data collection

**Table:**

Bruker Kappa APEX CCD area detector diffractometer	12260 independent reflections
Radiation source: sealed tube	12121 reflections with *I* > 2σ(*I*)
Triumph monochromator	*R*_int_ = 0.050
Detector resolution: 8 pixels mm^-1^	θ_max_ = 32.7°, θ_min_ = 1.6°
ω and φ scans	*h* = −15→15
Absorption correction: multi-scan (SADABS; Bruker, 2016)	*k* = −15→15
*T*_min_ = 0.559, *T*_max_ = 0.746	*l* = −38→39
148130 measured reflections	

### Tris(2,2'-bipyridine-κ2N,N')copper(II) bis[hexafluoridotantalate(V)] (III) Refinement

**Table:**

Refinement on *F*^2^	H-atom parameters constrained
Least-squares matrix: full	*w* = 1/[σ^2^(*F*_o_^2^) + (0.0106*P*)^2^ + 0.1438*P*] where *P* = (*F*_o_^2^ + 2*F*_c_^2^)/3
*R*[*F*^2^ > 2σ(*F*^2^)] = 0.016	(Δ/σ)_max_ = 0.002
*wR*(*F*^2^) = 0.031	Δρ_max_ = 0.96 e Å^−^^3^
*S* = 1.03	Δρ_min_ = −0.85 e Å^−^^3^
12260 reflections	Extinction correction: SHELXL-2018/3 (Sheldrick 2015b), Fc^*^=kFc[1+0.001xFc^2^λ^3^/sin(2θ)]^-1/4^
462 parameters	Extinction coefficient: 0.00031 (3)
1 restraint	Absolute structure: Flack *x* determined using 5861 quotients [(*I*^+^)-(*I*^-^)]/[(*I*^+^)+(*I*^-^)] (Parsons *et al.*, 2013)
Hydrogen site location: inferred from neighbouring sites	Absolute structure parameter: 0.5036 (7)

### Tris(2,2'-bipyridine-κ2N,N')copper(II) bis[hexafluoridotantalate(V)] (III) Special details

**Table:**

Geometry. All esds (except the esd in the dihedral angle between two l.s. planes) are estimated using the full covariance matrix. The cell esds are taken into account individually in the estimation of esds in distances, angles and torsion angles; correlations between esds in cell parameters are only used when they are defined by crystal symmetry. An approximate (isotropic) treatment of cell esds is used for estimating esds involving l.s. planes.
Refinement. Refined as a 2-component twin.

### Tris(2,2'-bipyridine-κ2N,N')copper(II) bis[hexafluoridotantalate(V)] (III) Fractional atomic coordinates and isotropic or equivalent isotropic displacement parameters (Å^2^)

**Table:**

	*x*	*y*	*z*	*U*_iso_*/*U*_eq_	
Ta2	0.00684 (3)	−0.03219 (2)	0.32198 (2)	0.01774 (4)	
Ta1	0.70686 (2)	1.36139 (2)	0.47151 (2)	0.01986 (4)	
Cu1	0.32411 (6)	0.64362 (5)	0.57036 (2)	0.01228 (8)	
F2	0.5183 (3)	1.2403 (5)	0.44310 (13)	0.0633 (12)	
F4	0.7724 (4)	1.2402 (3)	0.44119 (10)	0.0408 (7)	
C17	−0.0981 (5)	0.5633 (6)	0.6138 (2)	0.0188 (9)	
H17	−0.153152	0.611150	0.608263	0.023*	
F8	−0.1759 (3)	−0.0847 (3)	0.35333 (9)	0.0300 (6)	
N4	0.1166 (4)	0.5474 (4)	0.59777 (12)	0.0126 (6)	
F5	0.9006 (3)	1.4788 (3)	0.49516 (10)	0.0347 (6)	
F12	0.0952 (3)	0.0141 (3)	0.38681 (9)	0.0402 (7)	
F6	0.6515 (4)	1.2435 (3)	0.53047 (10)	0.0494 (9)	
C16	0.0368 (5)	0.6141 (5)	0.59049 (14)	0.0130 (8)	
C18	−0.1519 (4)	0.4417 (5)	0.64542 (14)	0.0220 (7)	
H18	−0.243559	0.406714	0.662085	0.026*	
F9	−0.0912 (3)	−0.0756 (3)	0.25849 (8)	0.0268 (5)	
N3	0.2459 (3)	0.8080 (3)	0.54816 (11)	0.0142 (5)	
F3	0.7575 (3)	1.4716 (3)	0.41088 (10)	0.0428 (7)	
F10	−0.0341 (3)	−0.2286 (3)	0.32783 (10)	0.0295 (6)	
F11	0.1857 (3)	0.0098 (3)	0.29071 (10)	0.0305 (6)	
F7	0.0551 (3)	0.1672 (3)	0.31516 (9)	0.0283 (5)	
F1	0.6482 (4)	1.4867 (4)	0.50135 (13)	0.0455 (8)	
C15	0.1004 (4)	0.7442 (4)	0.55624 (12)	0.0138 (6)	
C19	−0.0715 (5)	0.3711 (4)	0.65269 (15)	0.0194 (7)	
H19	−0.107200	0.286719	0.673773	0.023*	
N2	0.5147 (4)	0.7648 (4)	0.53243 (12)	0.0135 (7)	
N5	0.4063 (3)	0.7268 (3)	0.64157 (10)	0.0152 (5)	
C9	0.7449 (6)	0.9811 (6)	0.51576 (17)	0.0206 (10)	
H9	0.823892	1.071865	0.527435	0.025*	
C6	0.5147 (4)	0.7167 (4)	0.48464 (13)	0.0138 (6)	
C23	0.5294 (5)	0.8446 (5)	0.73468 (18)	0.0230 (10)	
H23	0.573816	0.885667	0.766493	0.028*	
C24	0.5230 (4)	0.7175 (5)	0.71745 (14)	0.0216 (8)	
H24	0.562435	0.670166	0.737466	0.026*	
C10	0.6291 (5)	0.8936 (5)	0.54776 (16)	0.0171 (9)	
H10	0.630309	0.925531	0.581658	0.021*	
C22	0.4708 (5)	0.9115 (4)	0.70528 (14)	0.0216 (7)	
H22	0.471689	0.997549	0.716782	0.026*	
C11	0.3105 (5)	0.9276 (4)	0.51831 (14)	0.0180 (7)	
H11	0.413500	0.974026	0.512970	0.022*	
C21	0.4108 (4)	0.8499 (4)	0.65868 (14)	0.0191 (8)	
H21	0.371299	0.895865	0.637984	0.023*	
C20	0.0634 (4)	0.4284 (4)	0.62794 (13)	0.0157 (7)	
H20	0.119861	0.381628	0.632587	0.019*	
N1	0.2704 (3)	0.5298 (3)	0.50323 (10)	0.0130 (5)	
C5	0.3856 (4)	0.5755 (4)	0.47127 (12)	0.0135 (6)	
C7	0.6286 (4)	0.7992 (4)	0.45095 (14)	0.0205 (8)	
H7	0.627957	0.763870	0.417631	0.025*	
N6	0.3922 (4)	0.4832 (4)	0.60312 (12)	0.0180 (6)	
C26	0.4437 (4)	0.5196 (4)	0.65109 (13)	0.0164 (6)	
C8	0.7437 (5)	0.9345 (5)	0.46684 (15)	0.0240 (8)	
H8	0.820854	0.994029	0.444011	0.029*	
C13	0.0833 (4)	0.9178 (4)	0.50245 (13)	0.0196 (7)	
H13	0.027481	0.954984	0.486504	0.023*	
C25	0.4586 (4)	0.6591 (4)	0.67064 (13)	0.0150 (6)	
C14	0.0144 (4)	0.7946 (4)	0.53329 (13)	0.0179 (7)	
H14	−0.088668	0.745881	0.538648	0.022*	
C12	0.2327 (4)	0.9860 (4)	0.49499 (14)	0.0185 (7)	
H12	0.281469	1.071236	0.474305	0.022*	
C27	0.4749 (5)	0.4294 (5)	0.68023 (15)	0.0241 (9)	
H27	0.510287	0.456347	0.714018	0.029*	
C2	0.1346 (5)	0.3167 (4)	0.45214 (16)	0.0225 (8)	
H2	0.045309	0.228062	0.445925	0.027*	
C1	0.1471 (4)	0.4030 (4)	0.49352 (14)	0.0174 (7)	
H1	0.065657	0.371517	0.515813	0.021*	
C30	0.3758 (4)	0.3587 (4)	0.58279 (15)	0.0201 (7)	
H30	0.343309	0.335632	0.548553	0.024*	
C3	0.2530 (5)	0.3601 (4)	0.41977 (15)	0.0250 (8)	
H3	0.247427	0.300756	0.391649	0.030*	
C4	0.3810 (5)	0.4932 (4)	0.42942 (14)	0.0218 (8)	
H4	0.463614	0.526892	0.407591	0.026*	
C28	0.4537 (5)	0.2983 (5)	0.65946 (16)	0.0272 (9)	
H28	0.472935	0.234061	0.679147	0.033*	
C29	0.4043 (5)	0.2626 (5)	0.60978 (16)	0.0234 (8)	
H29	0.390410	0.174533	0.594640	0.028*	

### Tris(2,2'-bipyridine-κ2N,N')copper(II) bis[hexafluoridotantalate(V)] (III) Atomic displacement parameters (Å^2^)

**Table:**

	*U*^11^	*U*^22^	*U*^33^	*U*^12^	*U*^13^	*U*^23^
Ta2	0.01698 (8)	0.01449 (10)	0.01536 (7)	0.00308 (8)	−0.00052 (7)	0.00018 (7)
Ta1	0.01754 (9)	0.01929 (10)	0.01742 (7)	0.00521 (9)	0.00037 (7)	0.00145 (8)
Cu1	0.01235 (18)	0.0136 (2)	0.0094 (2)	0.00535 (17)	0.00046 (17)	−0.00043 (14)
F2	0.0201 (13)	0.077 (3)	0.0550 (18)	−0.0039 (17)	−0.0002 (12)	−0.019 (2)
F4	0.056 (2)	0.0339 (17)	0.0316 (14)	0.0220 (16)	0.0046 (14)	−0.0086 (12)
C17	0.018 (2)	0.019 (2)	0.0220 (19)	0.0108 (17)	0.0047 (18)	−0.0006 (18)
F8	0.0297 (12)	0.0280 (14)	0.0236 (11)	0.0079 (11)	0.0091 (9)	0.0002 (10)
N4	0.0142 (14)	0.0117 (15)	0.0106 (13)	0.0056 (12)	0.0025 (10)	0.0018 (11)
F5	0.0275 (12)	0.0382 (16)	0.0390 (14)	0.0169 (13)	−0.0148 (11)	−0.0190 (13)
F12	0.0493 (17)	0.0341 (15)	0.0229 (12)	0.0100 (14)	−0.0170 (12)	−0.0010 (11)
F6	0.074 (3)	0.0418 (16)	0.0303 (13)	0.0279 (18)	0.0220 (15)	0.0169 (12)
C16	0.0189 (19)	0.0114 (16)	0.0080 (16)	0.0071 (15)	0.0001 (13)	0.0001 (13)
C18	0.0182 (16)	0.0188 (18)	0.0262 (18)	0.0071 (16)	0.0076 (14)	0.0031 (16)
F9	0.0312 (12)	0.0345 (13)	0.0178 (10)	0.0188 (11)	−0.0044 (9)	−0.0056 (10)
N3	0.0130 (13)	0.0128 (14)	0.0144 (13)	0.0045 (11)	−0.0013 (11)	−0.0010 (11)
F3	0.0340 (17)	0.0616 (19)	0.0311 (13)	0.0226 (15)	0.0034 (13)	0.0225 (13)
F10	0.0232 (13)	0.0193 (11)	0.0406 (14)	0.0065 (10)	0.0020 (11)	0.0039 (11)
F11	0.0199 (12)	0.0248 (13)	0.0441 (15)	0.0091 (10)	0.0035 (11)	0.0046 (12)
F7	0.0343 (15)	0.0190 (11)	0.0296 (12)	0.0117 (11)	0.0006 (11)	−0.0009 (9)
F1	0.0452 (19)	0.052 (2)	0.0562 (18)	0.0374 (18)	0.0084 (15)	0.0021 (16)
C15	0.0157 (15)	0.0154 (15)	0.0112 (15)	0.0085 (13)	0.0001 (12)	−0.0002 (12)
C19	0.0216 (18)	0.0149 (16)	0.0203 (18)	0.0081 (15)	0.0078 (15)	0.0069 (14)
N2	0.0127 (14)	0.0116 (14)	0.0140 (14)	0.0045 (12)	−0.0019 (10)	−0.0008 (12)
N5	0.0169 (12)	0.0146 (13)	0.0135 (12)	0.0073 (11)	0.0019 (10)	0.0012 (10)
C9	0.017 (2)	0.018 (2)	0.022 (2)	0.0049 (17)	−0.0038 (17)	−0.0039 (18)
C6	0.0152 (14)	0.0142 (16)	0.0128 (14)	0.0079 (13)	0.0014 (11)	−0.0009 (13)
C23	0.026 (2)	0.025 (2)	0.0129 (19)	0.0091 (16)	−0.0027 (17)	−0.0043 (18)
C24	0.0247 (18)	0.025 (2)	0.0124 (16)	0.0105 (16)	−0.0056 (14)	−0.0019 (14)
C10	0.0132 (17)	0.020 (2)	0.0144 (18)	0.0050 (15)	−0.0033 (14)	−0.0005 (15)
C22	0.0217 (19)	0.0186 (15)	0.0183 (16)	0.0054 (16)	−0.0012 (16)	−0.0064 (13)
C11	0.0213 (18)	0.0138 (16)	0.0163 (18)	0.0070 (14)	0.0013 (14)	−0.0006 (13)
C21	0.025 (2)	0.0173 (15)	0.0147 (15)	0.0105 (15)	0.0009 (14)	−0.0006 (12)
C20	0.0182 (17)	0.0139 (15)	0.0159 (16)	0.0087 (14)	0.0017 (13)	0.0016 (13)
N1	0.0131 (13)	0.0134 (12)	0.0130 (12)	0.0069 (11)	−0.0012 (11)	−0.0007 (9)
C5	0.0149 (16)	0.0146 (16)	0.0107 (13)	0.0071 (12)	−0.0003 (12)	−0.0007 (12)
C7	0.0176 (16)	0.0226 (19)	0.0143 (16)	0.0047 (15)	0.0020 (13)	−0.0016 (14)
N6	0.0201 (16)	0.0217 (15)	0.0157 (14)	0.0129 (13)	0.0011 (12)	−0.0002 (12)
C26	0.0128 (16)	0.0210 (19)	0.0147 (15)	0.0078 (14)	0.0021 (14)	0.0025 (14)
C8	0.0173 (18)	0.023 (2)	0.0217 (19)	0.0022 (16)	0.0042 (14)	0.0046 (16)
C13	0.027 (2)	0.0222 (18)	0.0170 (15)	0.0175 (16)	0.0036 (14)	0.0070 (14)
C25	0.0143 (15)	0.0166 (16)	0.0115 (14)	0.0056 (13)	−0.0006 (12)	0.0000 (12)
C14	0.0193 (17)	0.0213 (18)	0.0181 (17)	0.0137 (15)	0.0038 (14)	0.0025 (14)
C12	0.0248 (19)	0.0169 (17)	0.0134 (17)	0.0102 (15)	0.0014 (15)	0.0042 (14)
C27	0.032 (2)	0.033 (2)	0.0150 (17)	0.022 (2)	−0.0067 (16)	−0.0010 (16)
C2	0.0179 (18)	0.0167 (16)	0.0276 (19)	0.0048 (16)	−0.0019 (16)	−0.0068 (14)
C1	0.0173 (17)	0.0141 (15)	0.0170 (16)	0.0050 (13)	0.0002 (14)	−0.0006 (13)
C30	0.0221 (18)	0.0204 (18)	0.0195 (17)	0.0118 (15)	−0.0023 (15)	−0.0033 (15)
C3	0.030 (2)	0.0204 (18)	0.0198 (18)	0.0088 (17)	−0.0024 (17)	−0.0088 (15)
C4	0.0222 (19)	0.0203 (18)	0.0161 (16)	0.0055 (15)	0.0037 (15)	−0.0032 (14)
C28	0.036 (2)	0.033 (2)	0.0241 (19)	0.025 (2)	−0.0039 (18)	0.0042 (18)
C29	0.027 (2)	0.0218 (19)	0.028 (2)	0.0166 (17)	0.0006 (16)	−0.0019 (16)

### Tris(2,2'-bipyridine-κ2N,N')copper(II) bis[hexafluoridotantalate(V)] (III) Geometric parameters (Å, º)

**Table:**

Ta2—F8	1.901 (2)	C23—C24	1.381 (7)
Ta2—F12	1.885 (2)	C23—C22	1.381 (6)
Ta2—F9	1.894 (2)	C24—H24	0.9500
Ta2—F10	1.893 (3)	C24—C25	1.391 (5)
Ta2—F11	1.892 (3)	C10—H10	0.9500
Ta2—F7	1.903 (2)	C22—H22	0.9500
Ta1—F2	1.894 (3)	C22—C21	1.382 (5)
Ta1—F4	1.901 (3)	C11—H11	0.9500
Ta1—F5	1.883 (2)	C11—C12	1.388 (5)
Ta1—F6	1.886 (3)	C21—H21	0.9500
Ta1—F3	1.884 (2)	C20—H20	0.9500
Ta1—F1	1.883 (3)	N1—C5	1.350 (4)
Cu1—N4	2.024 (3)	N1—C1	1.341 (5)
Cu1—N3	2.330 (3)	C5—C4	1.385 (5)
Cu1—N2	2.020 (3)	C7—H7	0.9500
Cu1—N5	2.064 (3)	C7—C8	1.394 (5)
Cu1—N1	2.047 (3)	N6—C26	1.350 (5)
Cu1—N6	2.305 (3)	N6—C30	1.343 (5)
C17—H17	0.9500	C26—C25	1.487 (5)
C17—C16	1.384 (6)	C26—C27	1.380 (5)
C17—C18	1.386 (6)	C8—H8	0.9500
N4—C16	1.350 (6)	C13—H13	0.9500
N4—C20	1.344 (5)	C13—C14	1.386 (5)
C16—C15	1.488 (5)	C13—C12	1.377 (5)
C18—H18	0.9500	C14—H14	0.9500
C18—C19	1.389 (6)	C12—H12	0.9500
N3—C15	1.346 (5)	C27—H27	0.9500
N3—C11	1.343 (5)	C27—C28	1.393 (6)
C15—C14	1.393 (5)	C2—H2	0.9500
C19—H19	0.9500	C2—C1	1.380 (5)
C19—C20	1.394 (5)	C2—C3	1.384 (6)
N2—C6	1.354 (5)	C1—H1	0.9500
N2—C10	1.348 (6)	C30—H30	0.9500
N5—C21	1.349 (4)	C30—C29	1.385 (5)
N5—C25	1.336 (4)	C3—H3	0.9500
C9—H9	0.9500	C3—C4	1.397 (6)
C9—C10	1.384 (7)	C4—H4	0.9500
C9—C8	1.374 (6)	C28—H28	0.9500
C6—C5	1.469 (5)	C28—C29	1.386 (6)
C6—C7	1.390 (5)	C29—H29	0.9500
C23—H23	0.9500		
			
F8—Ta2—F7	92.03 (12)	C7—C6—C5	123.4 (3)
F12—Ta2—F8	88.82 (12)	C24—C23—H23	120.3
F12—Ta2—F9	176.22 (13)	C22—C23—H23	120.3
F12—Ta2—F10	91.26 (13)	C22—C23—C24	119.5 (4)
F12—Ta2—F11	91.61 (13)	C23—C24—H24	120.2
F12—Ta2—F7	88.58 (12)	C23—C24—C25	119.7 (4)
F9—Ta2—F8	87.85 (10)	C25—C24—H24	120.2
F9—Ta2—F7	89.75 (11)	N2—C10—C9	122.2 (4)
F10—Ta2—F8	90.12 (12)	N2—C10—H10	118.9
F10—Ta2—F9	90.53 (11)	C9—C10—H10	118.9
F10—Ta2—F7	177.84 (12)	C23—C22—H22	120.9
F11—Ta2—F8	177.08 (12)	C23—C22—C21	118.2 (4)
F11—Ta2—F9	91.81 (11)	C21—C22—H22	120.9
F11—Ta2—F10	86.98 (12)	N3—C11—H11	118.7
F11—Ta2—F7	90.87 (12)	N3—C11—C12	122.6 (4)
F2—Ta1—F4	89.58 (16)	C12—C11—H11	118.7
F5—Ta1—F2	175.49 (14)	N5—C21—C22	122.2 (4)
F5—Ta1—F4	86.68 (13)	N5—C21—H21	118.9
F5—Ta1—F6	92.12 (14)	C22—C21—H21	118.9
F5—Ta1—F3	89.74 (13)	N4—C20—C19	122.5 (4)
F6—Ta1—F2	90.39 (16)	N4—C20—H20	118.7
F6—Ta1—F4	90.02 (13)	C19—C20—H20	118.7
F3—Ta1—F2	87.64 (16)	C5—N1—Cu1	113.1 (2)
F3—Ta1—F4	88.41 (14)	C1—N1—Cu1	126.3 (2)
F3—Ta1—F6	177.48 (15)	C1—N1—C5	119.0 (3)
F1—Ta1—F2	92.03 (18)	N1—C5—C6	115.0 (3)
F1—Ta1—F4	178.14 (16)	N1—C5—C4	121.8 (3)
F1—Ta1—F5	91.67 (14)	C4—C5—C6	123.2 (3)
F1—Ta1—F6	90.91 (14)	C6—C7—H7	120.5
F1—Ta1—F3	90.72 (14)	C6—C7—C8	118.9 (3)
N4—Cu1—N3	76.58 (13)	C8—C7—H7	120.5
N4—Cu1—N5	90.44 (12)	C26—N6—Cu1	111.6 (2)
N4—Cu1—N1	95.81 (13)	C30—N6—Cu1	129.0 (3)
N4—Cu1—N6	98.80 (14)	C30—N6—C26	119.1 (3)
N2—Cu1—N4	166.58 (14)	N6—C26—C25	115.6 (3)
N2—Cu1—N3	90.89 (13)	N6—C26—C27	121.5 (4)
N2—Cu1—N5	96.14 (12)	C27—C26—C25	122.8 (3)
N2—Cu1—N1	80.79 (13)	C9—C8—C7	119.5 (4)
N2—Cu1—N6	94.16 (13)	C9—C8—H8	120.2
N5—Cu1—N3	98.11 (11)	C7—C8—H8	120.2
N5—Cu1—N6	75.75 (12)	C14—C13—H13	120.1
N1—Cu1—N3	96.98 (11)	C12—C13—H13	120.1
N1—Cu1—N5	164.65 (12)	C12—C13—C14	119.8 (3)
N1—Cu1—N6	89.41 (11)	N5—C25—C24	120.7 (4)
N6—Cu1—N3	172.42 (10)	N5—C25—C26	117.4 (3)
C16—C17—H17	120.4	C24—C25—C26	121.9 (3)
C16—C17—C18	119.3 (5)	C15—C14—H14	120.9
C18—C17—H17	120.4	C13—C14—C15	118.3 (4)
C16—N4—Cu1	119.0 (3)	C13—C14—H14	120.9
C20—N4—Cu1	121.2 (3)	C11—C12—H12	120.7
C20—N4—C16	119.3 (3)	C13—C12—C11	118.6 (3)
C17—C16—C15	121.7 (4)	C13—C12—H12	120.7
N4—C16—C17	121.3 (4)	C26—C27—H27	120.4
N4—C16—C15	117.0 (4)	C26—C27—C28	119.1 (4)
C17—C18—H18	120.0	C28—C27—H27	120.4
C17—C18—C19	119.9 (4)	C1—C2—H2	120.2
C19—C18—H18	120.0	C1—C2—C3	119.6 (4)
C15—N3—Cu1	109.0 (2)	C3—C2—H2	120.2
C11—N3—Cu1	129.8 (3)	N1—C1—C2	122.0 (4)
C11—N3—C15	118.4 (3)	N1—C1—H1	119.0
N3—C15—C16	115.5 (3)	C2—C1—H1	119.0
N3—C15—C14	122.3 (3)	N6—C30—H30	118.7
C14—C15—C16	122.2 (4)	N6—C30—C29	122.6 (4)
C18—C19—H19	121.2	C29—C30—H30	118.7
C18—C19—C20	117.7 (3)	C2—C3—H3	120.8
C20—C19—H19	121.2	C2—C3—C4	118.4 (4)
C6—N2—Cu1	114.0 (3)	C4—C3—H3	120.8
C10—N2—Cu1	126.3 (3)	C5—C4—C3	119.1 (4)
C10—N2—C6	119.1 (3)	C5—C4—H4	120.5
C21—N5—Cu1	121.0 (2)	C3—C4—H4	120.5
C25—N5—Cu1	119.3 (2)	C27—C28—H28	120.3
C25—N5—C21	119.7 (3)	C29—C28—C27	119.4 (4)
C10—C9—H9	120.5	C29—C28—H28	120.3
C8—C9—H9	120.5	C30—C29—C28	118.3 (4)
C8—C9—C10	118.9 (5)	C30—C29—H29	120.9
N2—C6—C5	115.3 (3)	C28—C29—H29	120.9
N2—C6—C7	121.3 (3)		

### catena-Poly[[diaqua(2,2'-bipyridine-κ2N,N')copper(II)]-µ-fluorido-tetrafluoridotin-µ-fluorido] (IV) Crystal data

**Table:**

[CuSnF_6_(C_10_H_8_N_2_)(H_2_O)_2_]	*F*(000) = 470
*M**_r_* = 488.45	*D*_x_ = 2.312 Mg m^−^^3^
Monoclinic, *P*2/*n*	Mo *K*α radiation, λ = 0.71073 Å
*a* = 6.2590 (2) Å	Cell parameters from 9773 reflections
*b* = 9.2167 (3) Å	θ = 2.2–37.7°
*c* = 12.1648 (3) Å	µ = 3.37 mm^−^^1^
β = 90.734 (2)°	*T* = 100 K
*V* = 701.70 (4) Å^3^	Block, blue
*Z* = 2	0.20 × 0.13 × 0.12 mm

### catena-Poly[[diaqua(2,2'-bipyridine-κ2N,N')copper(II)]-µ-fluorido-tetrafluoridotin-µ-fluorido] (IV) Data collection

**Table:**

Rigaku Oxford Diffraction XtaLAB Synergy, Single source at offset/far, HyPix diffractometer	3686 independent reflections
Radiation source: micro-focus sealed X-ray tube, PhotonJet (Mo) X-ray Source	3251 reflections with *I* > 2σ(*I*)
Mirror monochromator	*R*_int_ = 0.055
Detector resolution: 10.0000 pixels mm^-1^	θ_max_ = 38.2°, θ_min_ = 2.2°
ω scans	*h* = −10→10
Absorption correction: gaussian CrysalisPro (Rigaku OD, 2020)	*k* = −15→15
*T*_min_ = 0.732, *T*_max_ = 1.000	*l* = −20→21
22131 measured reflections	

### catena-Poly[[diaqua(2,2'-bipyridine-κ2N,N')copper(II)]-µ-fluorido-tetrafluoridotin-µ-fluorido] (IV) Refinement

**Table:**

Refinement on *F*^2^	Primary atom site location: dual
Least-squares matrix: full	Hydrogen site location: mixed
*R*[*F*^2^ > 2σ(*F*^2^)] = 0.029	H atoms treated by a mixture of independent and constrained refinement
*wR*(*F*^2^) = 0.067	*w* = 1/[σ^2^(*F*_o_^2^) + (0.0285*P*)^2^ + 0.1262*P*] where *P* = (*F*_o_^2^ + 2*F*_c_^2^)/3
*S* = 1.07	(Δ/σ)_max_ < 0.001
3686 reflections	Δρ_max_ = 2.05 e Å^−^^3^
110 parameters	Δρ_min_ = −0.85 e Å^−^^3^
0 restraints	

### catena-Poly[[diaqua(2,2'-bipyridine-κ2N,N')copper(II)]-µ-fluorido-tetrafluoridotin-µ-fluorido] (IV) Special details

**Table:**

Geometry. All esds (except the esd in the dihedral angle between two l.s. planes) are estimated using the full covariance matrix. The cell esds are taken into account individually in the estimation of esds in distances, angles and torsion angles; correlations between esds in cell parameters are only used when they are defined by crystal symmetry. An approximate (isotropic) treatment of cell esds is used for estimating esds involving l.s. planes.

### catena-Poly[[diaqua(2,2'-bipyridine-κ2N,N')copper(II)]-µ-fluorido-tetrafluoridotin-µ-fluorido] (IV) Fractional atomic coordinates and isotropic or equivalent isotropic displacement parameters (Å^2^)

**Table:**

	*x*	*y*	*z*	*U*_iso_*/*U*_eq_	
Sn1	0.500000	1.000000	0.500000	0.00931 (4)	
Cu1	0.250000	0.78803 (3)	0.750000	0.00868 (5)	
F1	0.42425 (17)	0.82052 (11)	0.57872 (8)	0.01353 (18)	
F2	0.78470 (17)	0.92222 (12)	0.46961 (9)	0.0184 (2)	
F3	0.39654 (19)	0.91649 (12)	0.36083 (8)	0.0183 (2)	
O1	0.4565 (2)	0.93265 (14)	0.80535 (10)	0.0147 (2)	
N1	0.0722 (2)	0.62522 (16)	0.69309 (12)	0.0136 (3)	
C5	0.1508 (4)	0.49153 (18)	0.71618 (16)	0.0184 (3)	
C1	−0.1085 (3)	0.6393 (3)	0.63416 (16)	0.0223 (4)	
H1	−0.162552	0.733418	0.618476	0.027*	
C4	0.0467 (5)	0.3679 (2)	0.6778 (2)	0.0339 (6)	
H4	0.103791	0.274557	0.693689	0.041*	
C2	−0.2186 (4)	0.5178 (3)	0.5954 (2)	0.0357 (6)	
H2	−0.347822	0.529058	0.554473	0.043*	
C3	−0.1386 (5)	0.3807 (3)	0.6169 (2)	0.0438 (8)	
H3	−0.210607	0.296937	0.590073	0.053*	
H1A	0.424 (6)	0.983 (3)	0.864 (3)	0.037 (9)*	
H1B	0.518 (6)	0.985 (3)	0.763 (3)	0.040 (10)*	

### catena-Poly[[diaqua(2,2'-bipyridine-κ2N,N')copper(II)]-µ-fluorido-tetrafluoridotin-µ-fluorido] (IV) Atomic displacement parameters (Å^2^)

**Table:**

	*U*^11^	*U*^22^	*U*^33^	*U*^12^	*U*^13^	*U*^23^
Sn1	0.01216 (7)	0.00790 (7)	0.00789 (6)	−0.00206 (4)	0.00114 (5)	0.00053 (4)
Cu1	0.00956 (11)	0.00682 (11)	0.00964 (11)	0.000	−0.00066 (9)	0.000
F1	0.0188 (5)	0.0100 (4)	0.0119 (4)	−0.0018 (3)	0.0028 (4)	0.0013 (3)
F2	0.0162 (5)	0.0176 (5)	0.0215 (5)	0.0034 (4)	0.0055 (4)	0.0071 (4)
F3	0.0284 (6)	0.0160 (5)	0.0103 (4)	−0.0102 (4)	−0.0013 (4)	−0.0008 (4)
O1	0.0188 (6)	0.0145 (6)	0.0110 (5)	−0.0075 (4)	0.0021 (4)	−0.0025 (4)
N1	0.0151 (6)	0.0130 (6)	0.0128 (6)	−0.0055 (5)	0.0019 (5)	−0.0024 (5)
C5	0.0299 (10)	0.0091 (7)	0.0166 (8)	−0.0056 (6)	0.0097 (7)	−0.0022 (5)
C1	0.0154 (8)	0.0346 (11)	0.0169 (8)	−0.0093 (7)	−0.0004 (6)	−0.0036 (7)
C4	0.0571 (16)	0.0153 (9)	0.0297 (11)	−0.0190 (9)	0.0126 (11)	−0.0078 (8)
C2	0.0306 (12)	0.0541 (16)	0.0223 (10)	−0.0294 (11)	0.0024 (9)	−0.0094 (10)
C3	0.0595 (18)	0.0433 (15)	0.0288 (11)	−0.0414 (14)	0.0116 (12)	−0.0150 (11)

### catena-Poly[[diaqua(2,2'-bipyridine-κ2N,N')copper(II)]-µ-fluorido-tetrafluoridotin-µ-fluorido] (IV) Geometric parameters (Å, º)

**Table:**

Sn1—F1	1.9723 (10)	O1—H1B	0.81 (3)
Sn1—F1^i^	1.9723 (10)	N1—C5	1.355 (2)
Sn1—F2	1.9603 (11)	N1—C1	1.337 (2)
Sn1—F2^i^	1.9603 (11)	C5—C5^ii^	1.481 (5)
Sn1—F3^i^	1.9621 (10)	C5—C4	1.390 (3)
Sn1—F3	1.9621 (10)	C1—H1	0.9500
Cu1—F1	2.3830 (10)	C1—C2	1.394 (3)
Cu1—F1^ii^	2.3830 (10)	C4—H4	0.9500
Cu1—O1^ii^	1.9695 (12)	C4—C3	1.374 (4)
Cu1—O1	1.9695 (12)	C2—H2	0.9500
Cu1—N1^ii^	1.9875 (14)	C2—C3	1.382 (4)
Cu1—N1	1.9875 (14)	C3—H3	0.9500
O1—H1A	0.88 (3)		
			
F1—Sn1—F1^i^	180.0	N1^ii^—Cu1—F1	97.98 (5)
F2—Sn1—F1^i^	89.45 (4)	N1—Cu1—F1	92.92 (5)
F2^i^—Sn1—F1	89.45 (4)	N1—Cu1—F1^ii^	97.98 (5)
F2^i^—Sn1—F1^i^	90.55 (4)	N1^ii^—Cu1—N1	81.95 (9)
F2—Sn1—F1	90.55 (4)	Sn1—F1—Cu1	130.20 (5)
F2^i^—Sn1—F2	180.0	Cu1—O1—H1A	118 (2)
F2—Sn1—F3	89.12 (5)	Cu1—O1—H1B	120 (3)
F2^i^—Sn1—F3	90.88 (5)	H1A—O1—H1B	109 (3)
F2^i^—Sn1—F3^i^	89.12 (5)	C5—N1—Cu1	114.47 (13)
F2—Sn1—F3^i^	90.88 (5)	C1—N1—Cu1	125.42 (14)
F3^i^—Sn1—F1^i^	90.64 (4)	C1—N1—C5	120.09 (17)
F3^i^—Sn1—F1	89.36 (4)	N1—C5—C5^ii^	114.51 (11)
F3—Sn1—F1^i^	89.36 (4)	N1—C5—C4	120.6 (2)
F3—Sn1—F1	90.64 (4)	C4—C5—C5^ii^	124.91 (16)
F3^i^—Sn1—F3	180.0	N1—C1—H1	119.5
F1—Cu1—F1^ii^	165.56 (5)	N1—C1—C2	121.0 (2)
O1^ii^—Cu1—F1^ii^	84.73 (5)	C2—C1—H1	119.5
O1—Cu1—F1	84.73 (5)	C5—C4—H4	120.0
O1^ii^—Cu1—F1	85.51 (5)	C3—C4—C5	119.9 (2)
O1—Cu1—F1^ii^	85.51 (5)	C3—C4—H4	120.0
O1—Cu1—O1^ii^	94.81 (8)	C1—C2—H2	120.2
O1—Cu1—N1	172.88 (6)	C3—C2—C1	119.6 (2)
O1^ii^—Cu1—N1^ii^	172.88 (6)	C3—C2—H2	120.2
O1—Cu1—N1^ii^	91.70 (6)	C4—C3—C2	118.8 (2)
O1^ii^—Cu1—N1	91.70 (6)	C4—C3—H3	120.6
N1^ii^—Cu1—F1^ii^	92.92 (5)	C2—C3—H3	120.6

Symmetry codes: (i) −*x*+1, −*y*+2, −*z*+1; (ii) −*x*+1/2, *y*, −*z*+3/2.

### catena-Poly[[diaqua(2,2'-bipyridine-κ2N,N')copper(II)]-µ-fluorido-tetrafluoridotin-µ-fluorido] (IV) Hydrogen-bond geometry (Å, º)

**Table:**

*D*—H···*A*	*D*—H	H···*A*	*D*···*A*	*D*—H···*A*
O1—H1*A*···F2^iii^	0.88 (3)	1.79 (3)	2.6444 (17)	165 (3)
O1—H1*B*···F3^i^	0.81 (3)	1.84 (4)	2.6293 (17)	164 (4)

Symmetry codes: (i) −*x*+1, −*y*+2, −*z*+1; (iii) *x*−1/2, −*y*+2, *z*+1/2.
